# Physiological Muscle Function Is Controlled by the Skeletal Endocannabinoid System in Murine Skeletal Muscles

**DOI:** 10.3390/ijms26115291

**Published:** 2025-05-30

**Authors:** Nyamkhuu Ganbat, Zoltán Singlár, Péter Szentesi, Elena Lilliu, Zoltán Márton Kohler, László Juhász, Anikó Keller-Pintér, Xaver Koenig, Fabio Arturo Iannotti, László Csernoch, Mónika Sztretye

**Affiliations:** 1 Department of Physiology, Faculty of Medicine, University of Debrecen, 4032 Debrecen, Hungary; 2 Doctoral School of Molecular Medicine, University of Debrecen, 4032 Debrecen, Hungary; 3 HUN-REN Cell Physiology Research Group, University of Debrecen, 4032 Debrecen, Hungary; 4 Department of Neurophysiology and Neuropharmacology, Center for Physiology and Pharmacology, Medical University of Vienna, 1090 Vienna, Austria; 5 Department of Biochemistry, Albert Szent-Györgyi Medical School, University of Szeged, 6720 Szeged, Hungary; 6 Institute of Surgical Research, Albert Szent-Györgyi Medical School, University of Szeged, 6720 Szeged, Hungary; 7 Institute of Biomolecular Chemistry (ICB), National Research Council of Italy (CNR), 80078 Pozzuoli, NA, Italy

**Keywords:** skeletal endocannabinoid system, murine skeletal muscle, in vivo muscle force, mitochondrial respiration, calcium homeostasis, store-operated calcium entry

## Abstract

The endocannabinoid system (ECS) is known to regulate crucial bodily functions, including healthy muscle activity. However, its precise roles in normal skeletal muscle function and the development of muscle disorders remain unclear. Previously, we developed a tamoxifen-inducible, skeletal muscle-specific CB_1_ receptor knockdown (skmCB1-KD) mouse model using the Cre/LoxP system. In this study, we aimed to clarify the mechanisms behind the observed reduction in muscle force generation in these mice. To investigate this, we analyzed calcium dynamics following electrical stimulation-induced muscle fatigue, assessed store-operated calcium entry (SOCE), and performed functional analysis of mitochondrial respiration. Our findings suggest that the reduced muscle performance observed in vivo likely arises from interconnected alterations in ATP production by mitochondria. Moreover, in skmCB1-KD mice, we detected a significant decrease in a component of the respiratory chain (complex IV) and a slowed dissipation of mitochondrial membrane potential upon the addition of an un-coupler (FCCP).

## 1. Introduction

Skeletal muscle, the largest organ in our body, comprises about 40 to 50% of total body mass and serves crucial roles for overall health, not only for maintaining posture and enabling mobility but also for regulating whole-body homeostasis and metabolism [1,2].

Muscle contraction underlying force generation is a result of a strict sequence of processes called excitation–contraction coupling (ECC) [3,4]. Any alteration of this well-orchestrated sequence of events could lead to altered force generation. During muscle contraction and force generation, chemical energy (ATP) is continuously synthesized and broken down into ADP and AMP. ATP, which acts as fuel for the cell through oxidative phosphorylation (OXPHOS), is produced by the mitochondria, a key organelle that occupies about 10–15% of the muscle fiber volume [5]. Mitochondria, these highly dynamic organelles that constantly undergo fission and fusion, are essential in decoding intracellular calcium (Ca^2+^) signals, thus contributing to the spatiotemporal distribution of intracellular Ca^2+^ concentrations [6,7,8,9]. Mitochondrial morphology is essential for ATP synthesis, and this dynamism allows for the maintenance of the organelle membrane potential (*ψ*_m_), which serves as the driving force for ATP synthesis within the electron transport chain (ETC).

Store-operated calcium entry (SOCE) is a Ca^2+^ influx pathway activated upon sarcoplasmic reticulum (SR) Ca^2+^ store depletion [10]. This process is based on the assembly and interaction of two proteins: stromal interaction molecule 1 (STIM1), a calcium sensor located in the SR membrane, and Orai1, a calcium channel located in the plasmalemma [11,12,13]. There are two distinct appearances of SOCE. The phasic (p)SOCE reflects the transient nature of the Ca^2+^ flux and is activated with every action potential when small Ca^2+^ depletions occur [14]. On the other hand, chronic (c)SOCE is slower and longer lasting and is activated following a partial (or virtually almost complete) SR depletion. This is either induced ex vivo through pharmacological activation of RyR1 or SERCA, or it occurs in vivo due to leaky RyR1s in pathological states like myopathies [15,16].

The endocannabinoid system (ECS) is a broadly distributed signaling network that is involved in a wide array of physiological processes such as mood and emotion, pain modulation, immune response, appetite, and metabolism [17,18,19,20]. The components of the system are the cannabinoid receptors (CB_1_R and CB_2_R), endocannabinoids (the two main ones are anandamide and 2-arachidonoyl-glycerol), and various enzymes and transport systems involved in their synthesis and degradation [21,22,23]. CB_1_Rs (encoded by the *Cnr1* gene) are the most abundant in muscle, being involved in cellular functions like autophagy [24], motor control and coordination [25], energy balance and metabolism [26,27], and exerting several biochemical effects, including ATP production and modulation of reactive oxygen species (ROS) [28]. In addition to CB_1_Rs located on the cell membrane, referred to as peripheral CB_1_ (pCB_1_R) [29,30], a population is present in muscle mitochondria (mtCB_1_R), where it regulates mitochondrial oxidative activity [31,32]. A recent study showed that the global deletion of CB_1_Rs induced a fast-to-slow-twitch fiber type conversion in mouse *M. gastrocnemius*, which increased its oxidative capacity and affected its antioxidant defense systems [33]. Antagonism of CB_1_ receptors using synthetic agents (e.g., AM6545 or rimonabant) has uncovered several interesting effects in preclinical studies using mice. Studies have shown that CB_1_ antagonism can increase glucose uptake and oxygen consumption in the isolated soleus muscle of obese mice, potentially contributing to improved blood sugar control [34]. Furthermore, it enhanced muscle protein synthesis and anabolism in a mouse model of dexamethasone-induced muscle atrophy [35]. Another study described improved muscle regeneration after injury following rimonabant usage [36]. *Cannabis sativa* contains various compounds called phytocannabinoids, which can interact with the ECS. The two most well known are Δ9-Tetrahydrocannabinol (THC), which is the primary psychoactive component that binds to both CB_1_ and CB_2_ receptors, acting as an agonist (activator). In human muscle, THC could potentially indirectly impact muscle function through central nervous system effects on motor control, coordination, and pain perception. Cannabidiol (CBD), another naturally occurring chemical compound found in the *Cannabis* plant, is non-psychoactive and has a more complex interaction with the ECS. Nevertheless, studies have shown that CBD acts as a negative allosteric modulator of CB_1_ receptors, potentially reducing the effects of THC and endocannabinoids at this receptor [37].

In this study, using a combination of genetic and pharmacological approaches, we aimed to unravel the intricate interplay between ECS signaling and skeletal muscle physiology using a muscle-specific CB_1_-KD mouse model. Our work sheds light on the role of ECS, specifically CB_1_Rs, in murine skeletal muscle, providing new insight into the mechanisms of ECS-mediated regulation of skeletal muscle function, force generation, mitochondrial energetics, and overall muscle contraction. This creates new opportunities for therapeutic approaches focused on enhancing muscle health, metabolism, and function.

## 2. Results

### 2.1. In Vivo Muscle Force and Motor Coordination Are Depressed in skmCB_1_-KD Mice

Our workgroup previously generated and characterized a tamoxifen-inducible skeletal muscle-specific CB_1_R knockdown (KD) mouse model (skmCB_1_-KD; [38]). However, a key question was left unaddressed: what was the mechanism behind the altered force production (both in vivo and in vitro) observed in these animals? Here, we decided to further investigate these aspects and tried to elucidate the causes and reveal the molecular mechanisms underlying altered muscle performance in skmCB_1_-KD mice.

**Figure 1:**
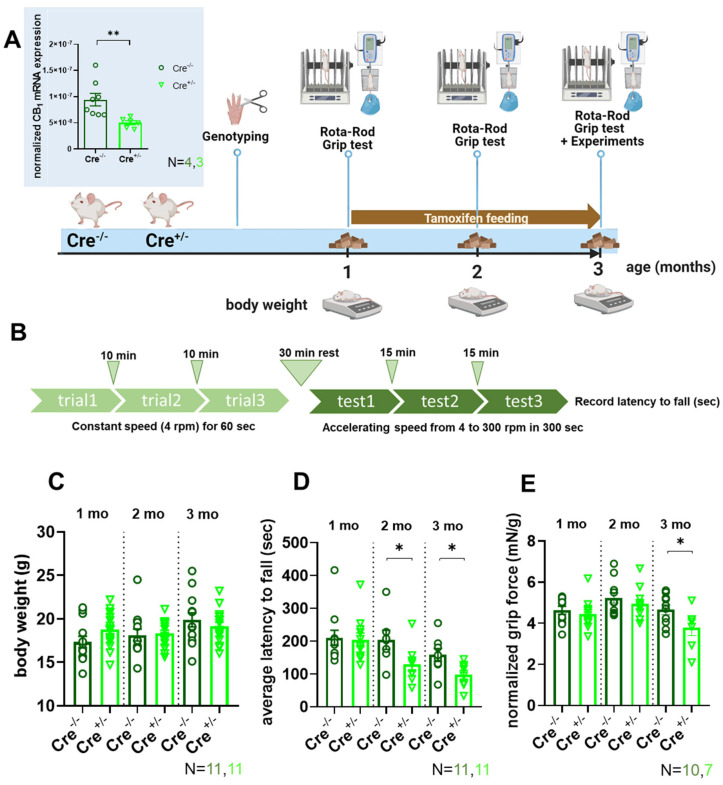
**In vivo grip force and coordination experiments.** (**A**) Experimental scheme for the evaluation of control (Cre^−/−^) and skmCB_1_-KD (Cre^+/−^) mice. Tamoxifen feeding was started at one month of age and lasted two months (scheme was created with BioRender). Inset shows the normalized CB_1_ mRNA expression measured via qPCR in *M. tibialis anterior* muscles. 18sRNA was used as housekeeping gene. n = 2 technical replicates from N = 4 and 3 biological samples for Cre^−/−^ (circles) and Cre^+/−^ (triangles), respectively. Here, and all subsequent cases (unless otherwise noted) Student’s *t*-test was used to assess significance. ** *p* < 0.009. (**B**) Rota-Rod measurements were used to assess muscle performance and coordination. The average latency to fall was recorded using the protocol illustrated. On the day of the evaluation 3 trials spaced at 1 min intervals were executed at a constant speed of 4 rpm that was maintained for 60 s for acclimatization purposes. After a 30 min’ rest period, the acceleration speed was gradually increased from 4 to 300 rpm in 5 min. Three test trials were recorded with 15 min intervals and the average of these trials was noted for each animal. (**C**) Body weight monitoring during tamoxifen feeding. For each mouse, the body weight was assessed weekly. The bar graph presents the average body weight starting from the beginning of the tamoxifen feeding at one month of age. N is the number of specimens investigated in each group (here, and all subsequent panels). (**D**) The average latency to fall was recorded at different age time points in Cre^−/−^ and Cre^+/−^ mice during the Rota-Rod test evaluations. Note that after one month of tamoxifen feeding, the Cre^+/−^ animals performed significantly worse, and this tendency was maintained until the end of feeding. * *p* ≤ 0.05. (**E**) Grip force normalized to body weight in Cre^−/−^ and Cre^+/−^ mice. After 2 months of tamoxifen feeding the Cre^+/−^ animals performed significantly worse than the control group. * *p* ≤ 0.05.

To induce skmCB_1_R-KD, we treated mice for 2 months with tamoxifen starting at one month of animal age (Figure 1A). The chosen tamoxifen treatment substantially reduces CB_1_R at mRNA and protein levels, as demonstrated in our earlier work [38]. The CB_1_ downregulation (on mRNA level) was verified in *M. tibialis anterior* samples (inset Figure 1A). Genotyping beforehand at 3 to 4 weeks of age allowed to assign animals into a Cre-recombinase negative (Cre^−/−^) and positive (Cre^+/−^) group, which served as control or allowed for skeletal muscle-specific tamoxifen-induced KD of CB_1_, respectively. Regular measurements of body weight, assessment of in vivo force using grip tests, as well as Rota-Rod evaluations (Figure 1A) were performed immediately before, 1, and 2 months after the initiation of tamoxifen treatment. The average weight of the mice was comparable across all tested time points before and after tamoxifen administration and did not differ between control (Cre^−/−^) and CB_1_R-KD (Cre^+/−^) mice (Figure 1C). To assess in vivo muscle performance and motor coordination, we measured the latency to fall for each mouse while the accelerating speed of the Rota-Rod was increased gradually from 4 to 300 rpm in 5 min (see the protocol in Figure 1B). The averaged data of these experiments clearly showed that already 1 month into the tamoxifen treatment the CB_1_R-KD (Cre^+/−^) mice performed worse, and their motor coordination was hindered (Figure 1D). While the latency to fall is a widely used parameter to evaluate motor performance, it is also influenced by motor coordination, balance and learning. As a second assessment of in vivo force production, we also performed measurements of the grip strength. When normalized to body weight the in vivo grip force was markedly declined at the end of the tamoxifen diet period in CB_1_R-KD mice compared to the control group (Figure 1E, 4.65 ± 0.23 mN/g for Cre^−/−^ vs. 3.77 ± 0.37* mN/g for Cre^+/−^, * *p* < 0.05). These data are in agreement with our previous findings (Figure 1H, from reference [38]).

### 2.2. SOCE Activity Is Preserved in skmCB_1_-KD Mice

We and others have shown earlier that store-operated calcium entry (SOCE) plays an important role in regulating fiber calcium content [14,39,40] and may thus contribute to the reduction in muscle function.

**Figure 2:**
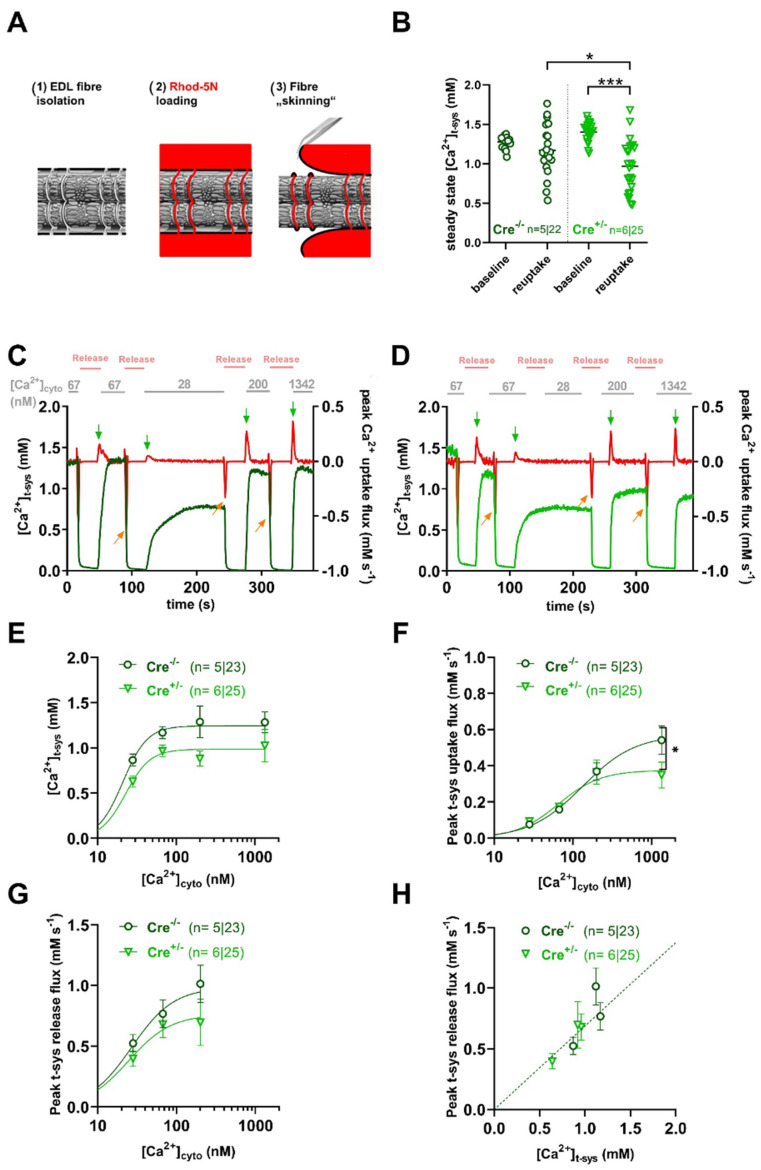
**Measurement of SOCE in skinned EDL fibers**. (**A**) Schematic representation of the protocol steps to perform measurements of SOCE in skinned fibers of mouse EDL muscles: (1) Isolated EDL fiber under paraffin oil with intact sarcolemma. (2) Incubation of the muscle fiber with a low-affinity Ca^2+^-sensitive dye (Rhod-5N). (3) Skinning of the fiber under paraffin oil traps Rhod-5N in the sealed t-system which allows measuring [Ca^2+^]_t-sys_ and enables full access to the cytoplasm. (**B**) The steady-state [Ca^2+^]_t-sys_ at baseline and after reuptake of Ca^2+^ following full depletion in Ca^2+^ and Mg^2+^ free and 30 mM caffeine solution (see panels C and D) in Cre^−/−^ and Cre^+/−^ mice. Note that in the Cre^+/−^ animals the reuptake was significantly smaller than in the Cre^−/−^ specimens. The numbers of animals/fibers are given in brackets. ANOVA with Tukey’s post hoc test was performed. * and *** indicate statistical significance at *p* < 0.05 and *p* < 0.001, respectively. (**C**,**D**) Typical recording of [Ca^2+^]_t-sys_ derived from the calibrated fluorescence of Rhod-5N in a control Cre^−/−^ (**C**) and a skmCB_1_-KD Cre^+/−^ (**D**) skinned fiber following steps 1–3 presented in panel A. The fiber is bathed in an internal solution containing 1 mM free Mg^2+^ and 67 nM free Ca^2+^. SOCE is induced through direct activation of the RyR1s by exposing the fiber to a “release” solution containing 0 mM free Mg^2+^ and 30 mM caffeine. Activation of pSOCE (dark and bright green traces) is seen as a steep depletion of [Ca^2+^]_t-sys_. The depletion is fully reversible as the t-system reloads with Ca^2+^ upon restoration of physiological [Ca^2+^]_cyto_ and [Mg^2+^]_cyto_. Red traces show the t-system release flux calculated as the first-time derivative of the dark or bright green traces. Green arrows indicate the peak t-system uptake fluxes at known calcium concentration content solutions. Orange arrows indicate the peak t-system release fluxes. (**E**–**G**) show [Ca^2+^]_t-sys_, the peak t-system release flux and the peak t-system uptake flux plotted as a function of [Ca^2+^]_cyto_. Note that the peak t-system uptake flux was altered at the highest concentration tested. *p  ≤  0.05. (**H**) Strong linear correlation between the peak t-system release flux (mMs^−1^) and [Ca^2+^]_t-sys_ (mM), but no difference between Cre^−/−^ and Cre^+/−^ fibers.

Here, we sought to investigate SOCE using the skinned fiber technique [41], which allows high-resolution measurement of t-system membrane Ca^2+^ fluxes, including SOCE [41,42]. Briefly, the t-tubular system of *M. extensor digitorum longus* (EDL) fibers from Cre^−/−^ and Cre^+/−^ mice was loaded with the low-affinity Ca^2+^-sensitive dye Rhod-5N before fiber skinning (Figure 2A). Calibrated measurement of Rhod-5N fluorescence from within the t-tubular system revealed a slight but significant difference in steady-state t-system Ca^2+^ ([Ca^2+^]_tsys_) under resting conditions when comparing Cre^−/−^ to Cre^+/−^ (compare columns 1 and 3 in Figure 2A). We then induced SOCE by activating the RyR1s via exposure of the fiber to a solution containing nominally 0 mM free Mg^2+^ and Ca^2+^, and 30 mM caffeine, respectively. Afterwards, we assessed the reuptake of Ca^2+^ into the t-system after re-exposing the fiber to a physiological free [Ca^2+^]_cyto_ of 67 nM. Steady-state Ca^2+^ levels after re-uptake were smaller compared to respective baseline values for both groups; however, the measured values turned out to be significantly smaller for Cre^+/−^ specimens (Figure 2B, columns 2 and 4). The pharmacological activation of SOCE and concomitant depletion of the t-system is fully reversible, such that the t-system reloads with Ca^2+^ upon restoration of physiological [Ca^2+^]_cyto_ and [Mg^2+^]_cyto_ values [43]. Such a sequence of depletion and reuptake was repeated at different [Ca^2+^]_cyto_ concentrations of 28, 67, 200, and 1342 nM for Cre^−/−^ and Cre^+/−^ fibers; original representative traces can be seen in Figure 2C,D, respectively. Evaluating [Ca^2+^]_tsys_ after re-uptake revealed consistently smaller (albeit non-significant) values in Cre^+/−^ compared to Cre^−/−^ for all Ca^2+^ concentrations tested (Figure 2E). However, peak t-system uptake flux was not altered under the same conditions (Figure 2F), except for the highest concentration tested. When we evaluated the SOCE flux determined as the peak t-system Ca^2+^ release flux upon 30 mM caffeine application in zero Ca^2+^/Mg^2+^conditions, we noticed a reduction (albeit non-significant) in the absolute values by trend (Figure 2G). The peak t-system fluxes derived in this manner are, however, dependent on the driving force between the t-system and the cytosol. Because [Ca^2+^]_t-sys_ was reduced in Cre^+/−^ fibers (Figure 2E), we therefore plotted respective flux values over the ambient steady state [Ca^2+^]_t-sys_ values immediately before depletion. A linear fit to these data is proportional to the t-system Ca^2+^ permeability and, as such, a relative measure of SOCE. Here, the linear relationship follows from the Goldman–Hodgkin–Katz current equation under the assumption of a sufficiently negative resting membrane potential close to −90 mV and a much larger Ca^2+^ concentration in the t-system compared to the cytosol [10] (see Methods section), two conditions that are readily met in the chosen preparation. When fitting respective data, both Cre^−/−^ and Cre^+/−^ samples showed a good linear correlation but were not different from each other (F-test; *p* = 0.77).

In agreement with SOCE being unaltered in CB_1_R-KD fibers, the protein expression of Orai1 and STIM1, the two key proteins thought to underlie SOCE in skeletal muscle [11,12,13], were likewise unaltered in Cre^+/−^ specimens when compared to Cre^−/−^ (Supplementary Figure S1A–C).

Collectively, these data suggest that the knockdown of CB_1_R did not alter SOCE but affected the ability of the fiber’s t-system to readily take up Ca^2+^ after depletion.

The t-system Ca^2+^-uptake proteins Na^+^/Ca^2+^ exchanger (NCX) and/or plasma membrane Ca^2+^ ATPase (PMCA) are known to equilibrate Ca^2+^ gradients across the t-system membrane in a bidirectional manner [43,44]. Ultimately, we found no changes in the expression of the latter on the protein level (Supplementary Figure S1D,E), suggesting that the observed reduction in t-system Ca^2+^-reuptake capacity may not relate to changes in these proteins but rather reflect changes at the level of the SR, such as altered SERCA or perhaps RyR1 function.

### 2.3. Calcium Homeostasis Is Preserved in skmCB_1_-KD Mice

Investigating a global CB_1_ knockout (KO) murine mouse model, our workgroup proposed earlier that CB_1_Rs are involved in the regulation of Ca^2+^ homeostasis of the skeletal muscle via a G_i_ protein and through a PKA-mediated mechanism [45]. Furthermore, our laboratory demonstrated that an immediate refilling of the SR following muscle activation would also require the proper functioning of SOCE [39].

**Figure 3:**
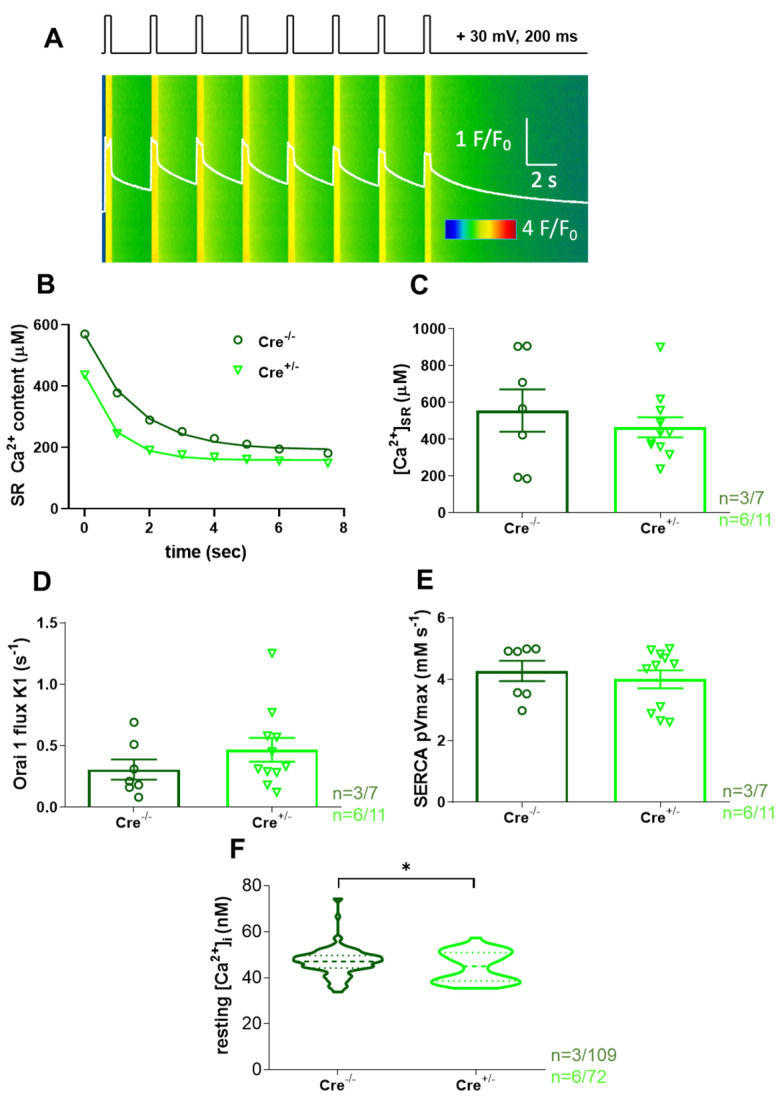
**Intracellular calcium homeostasis assessment in FDB fibers.** (**A**) Representative line-scan image of Rhod-2 fluorescence normalized to the baseline value F_0_(x) in a Cre^+/−^ FDB fiber subjected to successive rectangular depolarizing voltage steps (top black trace) under whole-cell voltage clamp. The Ca^2+^ transients were elicited by 200 ms long membrane depolarizations to +30 mV applied once per second. The white trace is the temporal profile of the normalized fluorescence (F) obtained by averaging 50 lines in the spatial domain normalized to average resting F_0_(x) values. (**B**) For one representative Cre^−/−^ and one Cre^+/−^ fiber the SR Ca^2+^ content was estimated from the amount released during each depolarizing pulse minus the removal flux. A single-exponential function was fitted to the points obtained following 8 consecutive stimuli (see Equation (A10) in Appendix A in Sztretye et al. [39]) with the following parameters: y_0_ = 190.7 vs. 158.7; a = 375.4 vs. 276.3; and b = 0.65 vs. 1.11, respectively, where b is the time constant of SR depletion, and a is the remaining SR calcium content after the last applied stimulus. (**C**) [Ca^2+^]_SR_ values were calculated for 7 and 11 FDB fibers from 3 Cre^−/−^ and 6 Cre^+/−^ animals, respectively. The calculation of [Ca^2+^]_SR_ was based on similar exponential fits as seen in (**B**). On average, by trend, in the Cre^+/−^ fibers (bright green), the SR calcium content was slightly lower than in the control Cre^−/−^ specimens. This difference was not statistically different. (**D**) Pooled data of the calculated fluxes through Orai1 channels (K1) as predicted by our previously introduced model (see Appendix A in Sztretye et al. [39]) show non-significant calcium flux changes upon CB_1_ knockdown in the Cre^+/−^ fibers. (**E**) The calculated SERCA pump activity (pVmax) is preserved in Cre^+/−^ fibers. (**F**) The resting intracellular calcium concentration is decreased following CB_1_R knockdown. Results presented as violin plots are from 109 and 72 fibers, from 3 Cre^−/−^ and 6 Cre^+/−^ animals, respectively. Dashed lines depict the median (central horizontal line in the box) while the dotted lines indicate the upper and lower quartiles. * indicates statistical significance at *p* < 0.03.

Thus, to specifically investigate the putative involvement of ECS in these processes within the skeletal muscles of the skmCB_1_-KD mouse model, we monitored the decay of the Ca^2+^ release following electrical stimulation in control (Cre^−/−^) and skmCB_1_-KD (Cre^+/−^) enzymatically dissociated *M. flexor digitorum brevis* (FDB) fibers. This was performed in a series of Ca^2+^ transients triggered by voltage depolarizations with the pattern illustrated in Figure 3A (top). In Figure 3A, the fluorescence transient depicted as F/F_0_ is shown as a white trace in a single FDB fiber isolated from a Cre^+/−^ mouse.

In Figure 3B, the SR Ca^2+^ content decline induced by voltage clamp stimulation was fitted with an exponential function for one representative Cre^−/−^ and one Cre^+/−^ FDB fiber. For the given examples, the SR Ca^2+^ decline at the end of the protocol was nearly identical in the two samples (190.7 µM for Cre^−/−^ vs. 158.7 µM for Cre^+/−^). One must note that the light green trace for Cre^+/−^ remains below the dark green Cre^−/−^ values, possibly suggesting a higher propensity for depletion and thus fatigue in the Cre^+/−^ animals. Overall, these data align with the SOCE experiments performed on skinned EDL fibers (Figure 2G). Nevertheless, the calculated [Ca^2+^]_SR_ was only reduced by trend in the Cre^+/−^ fibers and thus, unaffected by *Cnr1* genetic ablation (Figure 3C, 555.2 ± 114.9 µM for Cre^−/−^ vs. 464.7 ± 54.1 µM for Cre^+/−^).

In summary, our data suggest moderate fatigability, compromised t-system Ca^2+^ handling, and negligible changes in SOCE activity in skmCB_1_-KD Cre^+/−^ muscles. Our findings are in line with unaltered Orai1 flux values (K1 values, Figure 3D), as calculated with our previously introduced model.

The process of Ca^2+^ clearance from the cytosol in skeletal muscles is primarily accomplished by the SERCA pumps, which mediate the uptake of Ca^2+^ into the SR stores. In this work, the indirectly calculated SERCA pump activity was found to be preserved (Figure 3E) following CB_1_ downregulation (4.26 ± 0.32 mMs^−1^ for Cre^−/−^ vs. 3.99 ± 0.29 mMs^−1^ for Cre^+/−^).

Interestingly, when we measured the resting intracellular calcium concentration ([Ca^2+^]_i_) in FDB fibers loaded with the ratiometric dye Fura-2 AM, we found a small but significant reduction in Cre^+/−^ fibers (Figure 3F, 47.1 ± 0.6 nM for Cre^−/−^ vs. 44.9 ± 0.8* nM for Cre^+/−^, with * *p* < 0.03).

Taken together, our data suggest only minor alterations of Ca^2+^ homeostasis in CB_1_R-KD fibers. However, we believe these alterations might not be the (sole) reason behind the smaller in vivo grip force observed in this study and the decrease in vitro tetanic force demonstrated in our earlier work [38].

### 2.4. Mitochondrial Function Undergoes Modest Alterations in skmCB_1_-KD Mice

Mitochondria play a key role in maintaining skeletal muscle function, regulating oxidative stress, calcium homeostasis, and cellular respiration. Earlier, we proposed a role of CB_1_Rs and thus cannabinoid signaling in maintaining proper muscle function and mitochondrial morphology [38]. Here, we aimed to investigate whether the previously observed altered mitochondrial morphology following skeletal CB_1_R downregulation is also accompanied by changes in mitochondrial function. To test our hypothesis, we used high-resolution respirometry and two different protocols (short and long) to analyze potential changes in the mitochondrial respiration of *M. gastrocnemius* fibers isolated from the hind limbs of Cre^−/−^ and Cre^+/−^ mice.

**Figure 4:**
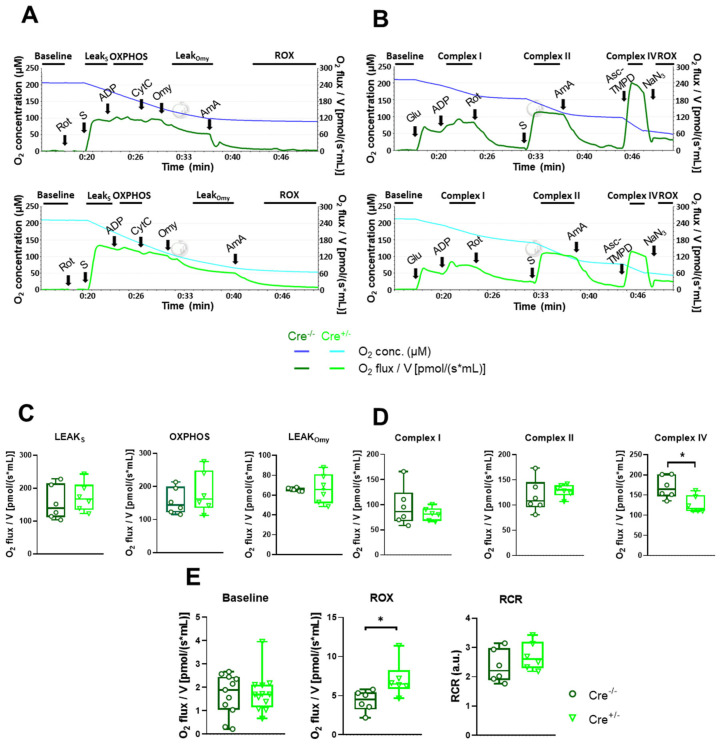
**Mitochondrial respiration measurement performed on *gastrocnemius* muscle fibers.** (**A**) Representative traces of the short treatment protocol for mitochondrial respiration measurement; the lines indicate the chamber O_2_ concentrations (blue and cyan) and O_2_ consumption (dark and bright green) in the Cre^−/−^ and Cre^+/−^ muscles samples, respectively. (**B**) Representative plots of the long treatment protocol; the lines show chamber O_2_ concentrations (blue and cyan) and O_2_ consumption (dark and bright green) in muscles from Cre^−/−^ and Cre^+/−^ mice. (**C**) Measured LEAK_S_ state, oxidative phosphorylation (OXPHOS), LEAK_Omy_ state are presented as O_2_ flux/V (pmol/[s × mL]). (**D**) Measured complex I-linked oxidative phosphorylation (complex I), complex II-linked oxidative phosphorylation (complex II), and complex IV respiratory activity (complex IV) are presented as O_2_ flux/V (pmol/[s × mL]). The box plots demonstrate the median (horizontal line in the box) and the 25th (lower whisker) and 75th (upper whisker) percentiles (n = 6). * indicates statistical significance at *p* < 0.01. (**E**) Baseline, residual oxygen consumption (ROX), and respiratory control ratio (RCR) are plotted as the ratio of OXPHOS and LEAK_Omy_ status. * indicates statistical significance at * *p* < 0.05. **Abbreviations:** Asc-TMPD, ascorbate and *N,N,N’,N’*-Tetramethyl-*p*-phenylenediamine dihydrochloride; AmA, antimycin A; au, arbitrary unit; CytC, cytochrome c; Glu, glutamate and malate; Omy, oligomycin; Rot, rotenone; S, succinate; NaN3, sodium azide.

First, we applied the short respiration protocol (Figure 4A). This allowed us to determine OXPHOS capacity and leak respiration before and after oligomycin (Omy) application, which inhibits ATP synthesis, resulting in a resting or unphosphorylated state (Figure 4C). Furthermore, routine or baseline respiration, residual oxygen consumption (ROX), and the respiratory control ratio (RCR) were also measured (Figure 4E). We did not observe any differences in these parameters upon CB_1_R knockdown. An exception was the increase in ROX (4.32 ± 0.54 pmol/s mL for Cre^−/−^ vs. 7.07 ± 0.92* pmol/s mL for Cre^+/−^, * *p* < 0.05), which was obtained after inhibition of the electron transport chain pathway. Various cellular enzymes that consume O_2_ and promote autoxidation reactions give rise to ROX, including peroxidase and oxidase activities that partially contribute to reactive oxygen species (ROS) production. The oxidative stress, thus ROS state at rest, was verified with the non-ratiometric fluorescent dye CellRox Green, but we did not find significant differences between the two specimens (Supplementary Figure S2).

Baseline and ROX were determined using the longer respiration protocol as well, along with OXPHOS linked to complex I and II and complex IV activity (Figure 4B). Among these functional values, only complex IV showed a change, specifically a significant decrease in Cre^+/−^ samples (Figure 4D, 169.7 ± 11.3 pmol/s mL vs. 126.8 ± 8.8* pmol/s mL, * *p* < 0.01). The respiratory control ratio (RCR) was unchanged (Figure 4E).

### 2.5. Mitochondrial Dynamics Marker Protein Levels Are Preserved in skmCB_1_-KD Mice

Since our previous [38] and current findings pointed towards morphological and functional mitochondrial alterations in skeletal muscles upon CB_1_R downregulation, we next decided to examine the expression level of proteins involved in the maintenance of mitochondrial calcium homeostasis and dynamics. Mitochondrial Ca^2+^ uptake is driven by the calcium-sensitive regulator MICU1, which modulates mitochondrial function by enhancing ATP production through the calcium-dependent activation of specific metabolic enzymes. Here, using molecular biology tools, we reconfirmed what we described in our prior work [38]: MICU1 was significantly downregulated on the protein level in Cre^+/−^ specimens. In the present work, we checked as well for the mitochondrial content of the voltage-dependent anion channel (VDAC), which was found unaffected by CB_1_ knockdown (Supplementary Figure S3A,C). These findings were paired with no detectable fiber type switch (at least on the mRNA level) (Supplementary Figure S4). Since no changes were seen on the mRNA level, this aspect was not investigated further.

We also examined mitochondrial fusion-related proteins: Mfn2, and Opa1 and found no changes, albeit a decline by trend in the latter could be identified (Supplementary Figure S5A,C,D). Drp1, a protein involved in fission processes and known to affect mitochondrial degradation, respiration, and ATP synthesis, showed a similar decline by trend following CB_1_ knockdown (Supplementary Figure S5A,B), but the change did not reach statistical significance.

### 2.6. FCCP-Dependent Dissipation of Mitochondrial Membrane Potential Is Delayed in skmCB_1_-KD Mice

The mitochondrial membrane potential (Δ*Ψ*_m_) generated by the proton pumps of complexes I, III, and IV is an essential component in the process of energy storage during oxidative phosphorylation, and it provides the driving force for ATP synthesis. Together with the proton gradient Δ*Ψ*_m_, it forms the transmembrane potential of hydrogen ions, which is harnessed to make ATP. Since our respirometry data shed light on a decline in the complex IV activity in Cre^+/−^ mice (Figure 4D), we were interested in exploring this aspect further.

**Figure 5:**
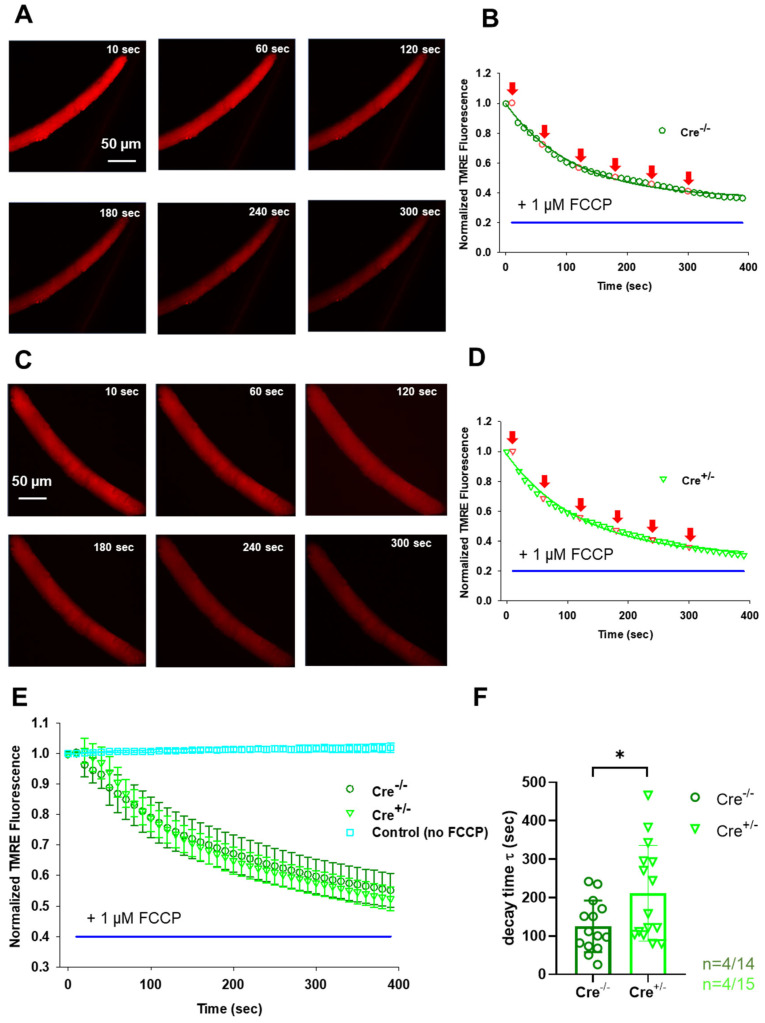
**Measurement of FCCP-dependent dissipation of mitochondrial membrane potential (*Δψ_m_*) in FDB fibers.** (**A**,**C**) Representative confocal image series of TMRE fluorescence recorded on an FDB fiber from a Cre^−/−^ (**A**) and a Cre^+/−^ (**C**) mouse, respectively, before (10 s) and during the application of 1 µM FCCP (indicated in panel (**B**,**D**) by the blue horizontal line). Note the fading over time of the dye fluorescence as the un-coupler poisons the organelle, leading to *Δψ_m_* loss. (**B,D**) Normalized TMRE fluorescence for the two representative cells presented in panels (**A**,**C**). The red symbols and red arrows indicate the time points when the image series shown in (**A**,**C**) were taken. The exponential fit (solid line) allowed for the calculation of *Δψ_m_* decay times (τ) for the two representative examples as follows: τ =111.7 s for Cre^−/−^ and τ =121.13 s for Cre^+/−^. (**E**) The averaged normalized TMRE fluorescence decay was slower in Cre^+/−^ mice. Data are plotted as means ±SEM. In cyan is the average of 5 control (C57/Bl6) FDB cells, where no FCCP was administered. (**F**) Averaged decay time values were significantly higher following FCCP administration in the skmCB_1_-KD specimens. * indicates statistical significance at * *p* < 0.02. **Abbreviations:** TMRE, tetramethylrhodamine, ethyl ester; FCCP, carbonyl cyanide-p-trifluoromethoxyphenylhydrazone.

To this end, we loaded single isolated FDB fibers from both strains with 20 nM TMRE—a dye that stains exclusively live mitochondria in the cell. While continuously scanning the fiber of interest with a confocal microscope, we followed the process of Δ*Ψ*_m_ dissipation upon the application of 1 µM FCCP, a well-known un-coupler of the electron transport chain (Figure 5A,B for Cre^−/−^ and Figure 5C,D for Cre^+/−^). Exponential fitting for individual cells (similar to those presented in panels B and D) was used to assess the decay time (τ) for n = 14 Cre^−/−^ and n = 15 Cre^+/−^ cells obtained from 4-4 animals, respectively. The obtained τ values were averaged and plotted for both groups (Figure 5E). Based on the individual exponential fits the average decay times were τ(Cre^−/−^) = 125.1 ± 17.9 s and τ(Cre^+/−^) = 211.3 ± 32.1* s, with * *p* < 0.02; Figure 5F), meaning that the normalized TMRE fluorescence dissipated slower in the Cre^+/−^ fibers. To exclude possible artifacts due to dye bleaching or leakage, we repeated the experiments in the absence of FCCP in 5 control cells from a wild-type C57Bl6 mouse, but essentially no signal loss was detected (Figure 5E, cyan symbols).

## 3. Discussion

In the present paper, we provide a thorough description of the role of CB_1_ receptors in murine skeletal muscles. We characterized the in vivo muscle grip force generation and motor coordination in skeletal muscle-specific CB_1_-KD mice (referred to as Cre^+/−^ throughout the manuscript) compared to littermate control Cre^−/−^ animals. Furthermore, we analyzed various aspects of cellular calcium homeostasis and mitochondrial respiration and function.

### 3.1. Muscle Force Production and Fiber Type Composition in skmCB_1_-KD Mice

When compared to control Cre^−/−^ mice, we found that Cre^+/−^ mice had reduced in vivo grip strength force and altered motor coordination (Figure 1). Gonzalez-Mariscal et al. [46] proposed that muscle-specific genetic ablation of CB_1_R influences muscle metabolism by decreasing fat accumulation, promoting oxidative phosphorylation, increasing muscle mass, and inducing a shift in muscle composition characterized by an enrichment of oxidative fibers. Ultimately, these changes were deemed to contribute to enhanced physical performance and an overall improvement in whole-body metabolism in CB_1_R knockout (KO) mice. Our current results about altered grip force development (including our earlier results on decline in vitro force production as described in Singlár et al. [38]) may seem somewhat contradictory to these findings. The main difference between the two studies might result from (a) the extent of CB_1_R knockdown; our inducible CB_1_R-KD model exhibits ~23% reduction in CB_1_ on the protein level. On the other hand, the study by González-Mariscal et al. [46] did not directly characterize CB_1_ protein expression but only the expression of Cre recombinase. Considering that their mouse model is a constitutive KO model, the degree of CB_1_ knockdown is presumably greater than 23%; (b) the study by González-Mariscal et al. used a constitutive CB_1_-KO model, where over time compensation mechanisms are likely to occur and influence overall muscle function [46].

Most of the muscles present a heterogeneous composition of slow- and fast-type fibers [47]. The spectrum of myosin heavy chain (MHC) isoforms is not fixed and can shift according to the following scheme: I↔IIa↔IIx↔IIb. Iannotti et al. reported that global CB_1_-KO mice have larger and more numerous muscle fibers compared to control animals [48]. Likewise, Senese et al. [33], working on the systemic CB_1_-KO model, described elevated oxidative stress and a marked enrichment of type I slow oxidative fibers upon global systemic lack of CB_1_ in *gastrocnemius* muscles. In our skmCB_1_-KD mice, however, we did not detect signs of fiber type switch when examining MHC isoforms on the mRNA level in *M. tibialis anterior* (TA) samples (a mixed composition muscle, Supplementary Figure S4).

### 3.2. Calcium Homeostasis in skmCB_1_-KD Mice

It is now well established that long-lasting stimulation of muscle fibers may result in the increase in cytoplasmic Ca^2+^ concentration accompanied by the reduction in SR Ca^2+^ release, SR store content, and enhanced ROS production. The metabolic changes in the working muscle (e.g., reduced ATP, increased ADP) may lead to changes in cellular Ca^2+^ homeostasis that contribute to physiological fatigue, as reduced Ca^2+^ release may eventually cause reduced force output. Alterations in [Ca^2+^] across organelles have been related to a variety of pathologies, where the persistent intracellular Ca^2+^ overload impairs ATP production, promotes excessive ROS production, and provokes the apoptotic cascade and mitochondrial damage. Interestingly, in our skmCB_1_-KD mice, we found significantly declined [Ca^2+^]_cyto_ values (Figure 3F), which would normally directly influence [Ca^2+^]_mito_ uptake as a compensatory mechanism to normalize the [Ca^2+^]_cyto_ [49]. We did not measure mitochondrial calcium here; however, in our earlier work, we did not identify signs of mitochondrial calcium overload (see Figure 6 from Singlár et al. [38]). In this study, changes in oxidative stress were observed through CellRox Green staining in FDB fibers from both specimens, but there was no evidence of excessive ROS production upon skmCB_1_ downregulation (Supplementary Figure S2). As discussed above, significantly lower levels of resting intracellular calcium were registered upon *Cnr1* genetic ablation in FDB fibers (Figure 3B). This may serve as an explanation for the lower calculated [Ca^2+^]_SR_ levels, which, however, did not result in enhanced SOCE activity as inferred from measurements in intact FDB (Figure 3D) and skinned EDL fibers (Figure 2). One feasible explanation for the minimal changes in SOCE could be the modest efficiency of CB_1_ knockdown induced by a 2-month-long tamoxifen diet. In the present work, we assessed the CB_1_ downregulation in TA muscles on the mRNA level (Figure 1A, inset). In our earlier work [38], we quantified in slow (SOL), fast (EDL), and mixed muscles (TA) the level of CB_1_ deficiency, and it was quite dramatic on the mRNA level (66.44%, 81.34%, and 66.04% for EDL, SOL, and TA), but more modest on the protein level (~23% in TA%). It remains elusive, however, whether an extended tamoxifen diet period would be more suitable to achieve a larger CB_1_ protein knockdown. During prolonged muscle stimulation (voltage or pharmacologically induced), it is speculated that pSOCE, in cooperation with the t-system Ca^2+^-uptake proteins Na^+^/Ca^2+^ exchanger (NCX) and/or plasma membrane Ca^2+^-ATPase (PMCA), equilibrates Ca^2+^ gradients across the t-system membrane in a bidirectional manner. These events lead to an influx of Ca^2+^ if [Ca^2+^]_cyto_ transients are lower and an efflux of Ca^2+^ if [Ca^2+^]_cyto_ transients are higher than “normal”. Moreover, [Ca^2+^]_cyto_ sets SR load and, according to Koenig et al. [50], also affects pSOCE. Here, we have analyzed the protein levels of the PMCA, and no alterations were detected when comparing the TA muscle samples from Cre^+/−^ and Cre^−/−^ specimens (Supplementary Figure S1D,E).

The decline in SR Ca^2+^ concentration due to voltage-induced electrical stimulation in FDB fibers was moderate in the skmCB1-KD Cre^+/−^ muscles (Figure 3B). This could indicate a slight impairment in SOCE dynamics within the Cre^+/−^ muscle fibers, potentially leading to a more significant depletion of Ca^2+^ from the SR (for details, see Equation (A10) from Sztretye et al. [39]). It was established that phasic SOCE depends on [Ca^2+^]_I_; thus, the intracellular calcium levels, albeit significantly decreased in Cre^+/−^ are only mildly reflected in the pSOCE measurements. Notably, the reduction in resting [Ca^2+^]_i_ observed upon *Cnr1* genetic depletion was modest, and we believe that the change is physiologically irrelevant, which may translate to insignificant changes in free Ca^2+^ concentration of the SR.

### 3.3. Mitochondria Function and Dynamics in skmCB_1_-KD Mice

In a working muscle, mitochondria will release ROS, which in turn could affect SOCE. Nevertheless, SOCE is completely controlled by the SR near-membrane Ca^2+^ content. The uptake of Ca^2+^ by the mitochondria is controlled by the [Ca^2+^]_cyto_, which is set by the Ca^2+^ permeability of the t-system (a balance of SOCE and PMCA activity [40,51]). On the other hand, more and more studies elaborate on the role of regulatory proteins that contribute to the connection between the sarco(endo)plasmic reticulum (SR) Ca^2+^ store and mitochondria contact sites referred to as mitochondria-associated membranes (MAMs). In mammalian skeletal muscle fibers, most mitochondria are in contact with the SR, providing a direct link between Ca^2+^ cycling and energy demand [52]. It is now generally accepted that mitochondria accumulate high [Ca^2+^] following physiological stimulation when [Ca^2+^]_cyto_ rises from 0.1 μM to about 2–3 μM. The SR holds ~90% of the fiber Ca^2+^ content at steady state. The mitochondria hold < 5% of the fiber Ca^2+^ content in healthy muscle [40], and until recently, it was not considered a significant candidate to sequester compelling amounts of Ca^2+^ during SR Ca^2+^ release. Nevertheless, the proximity between the SR and the mitochondria ensures the formation of high [Ca^2+^] microdomains, allowing mitochondria to rapidly take up Ca^2+^. For Ca^2+^ to enter the mitochondrial matrix, it requires the crossing of the outer and inner mitochondrial membranes (OMM and IMM). The initial passage across the permeable OMM occurs through the voltage-dependent anion channel (VDAC), which is highly expressed in the OMM. Therefore, the VDAC expression levels constrain the mitochondrial Ca^2+^ influx; overexpression of VDAC increases the mitochondrial Ca^2+^ uptake, whereas silencing VDAC reduces it [53]. Here, we have analyzed the protein levels of VDAC, which showed an almost 2-fold increase in Cre^+/−^ muscles (Supplementary Figure S4A,C). This increase, however, did not prove to be significant.

Furthermore, the accumulation of Ca^2+^ in the mitochondrial matrix occurs down to its electrochemical gradient via the mitochondrial calcium uniporter (MCU). MCU uses the electrochemical potential (Δ*Ψ*_m_ ~ −180 mV) established across the IMM to drive the Ca^2+^ entry into the matrix [54]. MICU1 protein, as part of the MCU holocomplex, acts as a gatekeeper, keeping the channel closed under resting conditions and establishing a Ca^2+^ threshold for mitochondrial uptake [55]. In a very recent study by Hasan et al. [56], MICU1/2 loss increased mitochondrial calcium influx and sensitized mitochondria to calcium overload injury. In our skmCB_1_-KD muscles, MICU1 levels were significantly decreased upon *Cnr1* genetic manipulation (Supplementary Figure S4B,D).

Treatment with protonophores like FCCP disrupts the Δ*Ψ*_m_, entirely abolishing the mitochondrial Ca^2+^ uptake process and depleting buffered mitochondrial Ca^2+^ [53,57]. In our experiments with FCCP, we observed significantly slower dissipation of Δ*Ψ*_m_ upon CB_1_ ablation in Cre^+/−^ muscles (Figure 5E,F). We propose that this phenomenon, along with decreased complex IV activity (Figure 4D), may contribute to a diminished ATP turnover and, consequently, a lower rate of energy production. This may explain the reduced in vivo grip force generation (as well as the reduced in vitro force findings presented by Singlár et al. [38]) and potentially lead to increased fatigability in Cre^+/−^ specimens.

Mitochondrial energy production is carried out by the five complexes inserted in the IMM. In summary, pyruvate, a by-product of glucose metabolism, enters the mitochondria through VDAC and is enzymatically converted into acetyl-CoA within the matrix. Further, the acetyl-CoA enters the Krebs cycle, where it undergoes oxidation, resulting in the production of NADH and FADH_2_ [58]. NADH is subsequently oxidized by complex I (NADH dehydrogenase), which transfers electrons to ubiquinone, reducing it to ubiquinol. Complex II (succinate dehydrogenase) is the only membrane-bound enzyme in the Krebs cycle, catalyzing the conversion of succinate to fumarate [59]. Complex III (ubiquinol-cytochrome C oxidoreductase) catalyzes the transport of H^+^ from the matrix to IS, coupled with the movement of electrons from ubiquinol to cytochrome C. After receiving the electrons, cytochrome C transports them to complex IV (cytochrome C oxidase). Lastly, complex IV then transfers the electrons to molecular oxygen, forming water. This process also drives the translocation of two protons (H⁺) from the matrix to the IS for each water molecule produced [60]. In our mitochondrial respiration experiments performed on freshly isolated *M. gastrocnemius* fibers, we found a marked decline in complex IV activity (Figure 4D), which could be associated with changes in organelle energy status, thus ATP provision. In this study, an examination of TA muscle fibers (Supplementary Figure S4), which have a mixed fiber composition similar to the *gastrocnemius* muscle used for the respirometry experiments, did not reveal any evidence of a fiber type shift towards an oxidative phenotype (at least at the mRNA level). Nevertheless, we cannot fully exclude this possibility.

CB_1_Rs have been proposed to regulate nitric oxide (NO) production through nitric oxide synthase (nNOS and eNOS); thus, their role has been associated with vascular health, cannabinoid-mediated neuroprotection, and mitochondrial biogenesis in adipose tissue and placenta [61,62]. Considering that NO is a direct inhibitor of complex IV, one cannot exclude the possibility that the regulation of NOS/NO production might also be involved in the impaired complex IV activity seen in CB_1_R-KD muscles. This hypothesis, however, was not verified in the present study.

Mitochondrial dynamics, essential for maintaining the proper shape and structure of mitochondria, rely on a fine-tuned equilibrium between division (fission) and fusion. This balance between fission and fusion events is crucial for healthy mitochondrial function. The proteins regulating fission are Drp1 and Fis1, whereas fusion is controlled by Mfn1, Mfn2, and Opa1. Mfn2 is located at both ER and mitochondrial membranes, where it has been reported to strengthen ER–mitochondria contacts and facilitate mitochondrial Ca^2+^ uptake [63,64]. Recently, it was proposed that Mfn2 acts as a negative regulator of ER-mitochondrial tethers, which, by reducing the number of MAM contacts, avoids toxic Ca^2+^ accumulation [65]. Earlier, we described significantly altered mitochondrial morphology upon muscle-specific CB_1_ downregulation [38]. In the present study, the mitochondrial dynamics-related proteins examined (Mfn2, Opa1, Drp1) displayed no expression level changes in Cre^+/−^ mice (Supplementary Figure S5).

### 3.4. CB_1_R-Mediated Signaling Pathways Supporting Skeletal Muscle Function

The CB_1_ receptor is a G protein-coupled receptor, characterized by its association with heterotrimeric G proteins (G_α_, G_β,_ and G_γ_ subunits). Formerly, our workgroup investigated a systemic CB_1_-KO mouse model and found that cannabinoid signaling inhibits sarcoplasmic Ca^2+^ release and regulates excitation–contraction coupling in mammalian skeletal muscles via a G_i_-PKA-mediated mechanism [45]. On the other hand, a few years ago, Iannotti et al. demonstrated that in C_2_C_12_ myoblasts, the effects of 2-AG or synthetic CB_1_ agonists were unaffected by pertussis toxin, indicating that CB_1_ coupling in these cells does not involve G_i_ proteins [48]. Instead, CB_1_ stimulation induced the hydrolysis of PIP_2_ (phosphatidylinositol 4,5-bisphosphate), which relies on G_q_-PLC-IP_3_ axis activation, thus elevating intracellular calcium concentration. Keeping in mind that the CB_1_ mRNA and protein expression profile during murine C_2_C_12_ myotube formation is highly time-sensitive, one possible explanation for the above-described discrepancy could be the experimental model studied: Oláh et al. [45] evaluated terminally differentiated C_2_C_12_ myotubes and adult FDB fibers from CB_1_-KO mice, whereas Iannotti et al. [48] examined differentiating C_2_C_12_ myoblasts.

Ultimately, we propose drawing an analogy with the CB_1_-KO model characterized by Oláh et al. [45]: in our skmCB_1_-KD mouse model, signaling mediated by the G_i/o_ protein through the inhibition of the adenylyl cyclase-cAMP-PKA axis may serve as a significant downstream effect of CB_1_ downregulation, potentially accounting for the altered aspects of calcium homeostasis presented in the present work (Figure 2 and Figure 3). However, we must acknowledge that we did not conduct experiments to confirm this hypothesis, and further investigations are needed to test this.

**Figure 6:**
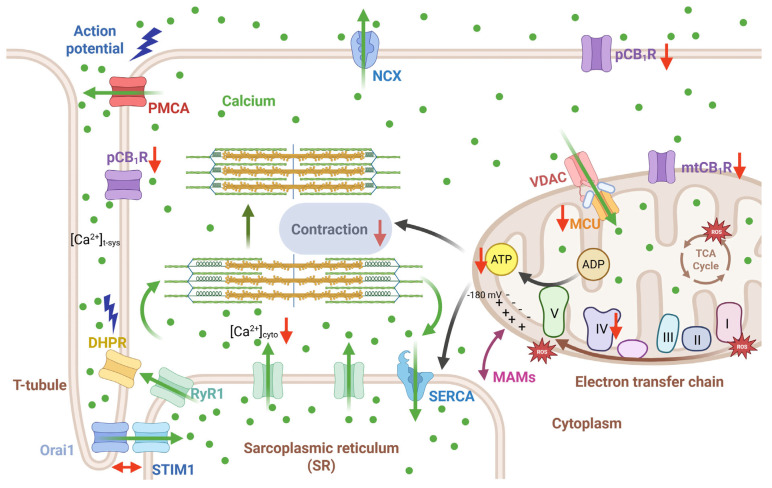
**Mitochondrial dysfunction underlies reduced muscle performance in CB_1_ receptor-deficient mice.** Red arrows indicate the changes obtained in our current experiments following tamoxifen-induced muscle-specific CB_1_ downregulation. Green arrows show the path followed by calcium ions. Briefly, during the ECC mechanism, calcium is released from the SR via the RyR1s and then binds to contractile proteins to induce contraction. SR depletion triggers SOCE activation via Orai1/STIM1 concerted action. Upon muscle relaxation, calcium is returned to the store via the SERCA pumps or sequestered by mitochondria via uptake by the VDAC/MCU proteins. Black arrows indicate the path followed by ATP to induce muscle contraction. Besides calcium, ATP is also critical for muscle contraction, and it underlies SERCA function as well. **Abbreviations:** pCB_1_R, plasma membrane cannabinoid receptor type 1; mtCB_1_R, mitochondrial cannabinoid receptor type 1; DHPR, dihydropyridine receptor; PMCA, plasma membrane calcium ATPase; Orai1, calcium release-activated calcium channel protein 1; STIM1, Stromal interaction molecule 1; RyR1, ryanodine receptor type 1; SERCA, sarco(endo)plasmic reticulum calcium ATPase; NCX, sodium calcium exchanger; ATP, adenosine trisphosphate; ADP, adenosine diphosphate; VDAC, voltage activated anion channel; MCU, mitochondrial calcium uniporter; ROS, reactive oxygen species; TCA, Tricarboxylic acid; MAM, mitochondria-associated membrane. Created in BioRender. Sztretye, M. (2025) https://BioRender.com.

Our research, summarized in Figure 6, reveals a substantial decrease in muscle contraction and in vivo force generation, concluding that this is likely due to impaired ATP production by the mitochondria. While there were modest shifts in intracellular calcium homeostasis, our findings suggest that CB_1_ receptors primarily impact ATP generation, not calcium directly. A crucial unanswered question remains: are these effects mediated by CB_1_ receptors located on the plasma membrane or within the mitochondria? This aspect requires further study. Nevertheless, building on our past work [38], we now have stronger evidence that CB_1_ receptors play a vital role in maintaining both muscle force and cellular/mitochondrial health. This understanding suggests that targeting CB_1_Rs could be a promising strategy to combat muscle weakness in various diseases, including sarcopenia and myopathies.

## 4. Materials and Methods

### 4.1. Animal Care

Animal experiments adhered to the guidelines of the European Community (86/609/EEC) and were designed to minimize animal suffering and distress. The experimental protocol was approved by the Institutional Animal Care Committee of the University of Debrecen (3-1/2019/DEMAB). The mice were housed in plastic cages with mesh covers and fed ad libitum with pelleted mouse chow and water. Room illumination was an automated cycle of 12 h light and 12 h dark, and the room temperature was maintained within the range of 22–25 °C.

#### 4.1.1. Generation of the Muscle-Specific CB_1_ Knockdown Mouse Strain

Tamoxifen-inducible muscle-specific CB_1_ knockdown mice were obtained as described in Singlár et al. [38]. Mixed-gender, tamoxifen-inducible, muscle-specific CB_1_ knockdown mice (hereinafter referred to as Cre^+/−^) and littermate controls (Cre^−/−^) were used here.

#### 4.1.2. Tamoxifen Diet

To induce CB_1_ ablation, a tamoxifen diet (per os) was started immediately after the separation of the Cre^+/−^ and Cre^−/−^ pups from the mother at the age of 4 weeks. Littermates were fed for 2 months without interruption (while monitoring normal body weight gain and performing grip strength and Rota-Rod tests; see Figure 1A) and then were used for the subsequent experiments. The tamoxifen-supplemented chow (Envigo, TD 130857) contained 500 mg tamoxifen/kg, providing 80 mg tamoxifen/kg body weight per day, assuming 20–25 g body weight and 3–4 g daily food intake.

### 4.2. Molecular Biology

#### 4.2.1. Genotyping

Genotyping was performed at 3 to 4 weeks of age, and the procedure helped to identify the animals and allowed their classification into experimental groups.

Total genomic DNA was isolated from finger biopsies and screened for the presence of the HSA-Cre recombinase cassette by PCR (Biometra Advanced Twin 48 G, 230 V, Analytik Jena GmbH, Jena, Germany) as described in detail earlier in Singlár et al. [38].

#### 4.2.2. Western Blot Analysis

*M. tibialis anterior* (TA) skeletal muscle tissues were homogenized in lysis buffer (20 mM Tris–HCl, 5 mM EGTA, Protease Inhibitor Cocktail (Sigma, St. Louis, MO, USA) with an HT Mini homogenizer (OPS Diagnostics, Lebanon, NJ, USA). Six-fold concentrated electrophoresis sample buffer (20 mM Tris–HCl, pH 7.4, 0.01% bromophenol blue dissolved in 10% SDS, 100 mM β-mercaptoethanol) was added to total lysates to adjust equal protein concentration of samples, and then the mixture was boiled for 5 min at 90 °C. Twenty micrograms of total protein was loaded into each lane and separated on a 10% SDS–polyacrylamide gel. Proteins were transferred to nitrocellulose membranes, blocked with 5% non-fat milk dissolved in phosphate saline buffer (PBS), and then the membranes were incubated with the appropriate primary antibodies overnight at 4 °C. The primary antibodies used for specific labeling are summarized in Table 1. After washing for 30 min in TBS supplemented with 1% Tween-20 (TBST), membranes were incubated with HRP-conjugated secondary antibodies (Blotting Grade Goat Anti-Rabbit IgG (H+L) (Human IgG Absorbed) Horseradish Peroxidase Conjugate (cat.no. 170-6515, Bio-Rad, Hercules, CA, USA) and Blotting Grade Affinity Purified Goat Anti-Mouse IgG (H+L) Horseradish Peroxidase Conjugate (cat.no. 170-6516). Membranes were developed, and signals were detected using enhanced chemiluminescence (Thermo Fisher Scientific, Waltham, MA, USA). Optical density of signals was measured by ImageJ software 1.53k (version Java 1.8.0_172, NIH, Bethesda, MD, USA), and results were normalized to the optical density of α-actinin (1:1000, cat.no. sc-166524, Santa Cruz Biotechnology, Dallas, TX, USA) and tubulin (1:4000, cat. no. T5168, Sigma, St. Louis, MO, USA), respectively (see Table 1).

#### 4.2.3. qPCR

TRI reagent (cat. no. TR118MRC, Cincinnati, OH, USA) was used to isolate total ribonucleic acid (RNA) fractions from homogenized skeletal muscle specimens of *M. flexor digitorum brevis* (FDB), *M. extensor digitorum longus* (EDL), *M. soleus* (SOL), and *M. tibialis anterior* (TA) from tamoxifen-fed Cre^+/−^ and Cre^−/−^ mice. Before use, the separated RNA samples were kept at −80 °C dissolved in nuclease-free water (NFW). A spectrophotometer set to 260 nm (NanoDrop ND1000; Promega Biosciences, Madison, WI, USA) was used to measure the RNA yield and purity. After treating the separated RNA samples with DNase and RNase inhibitor (Ambion, Austin, TX, USA), a High-Capacity cDNA Reverse Transcription Kit (cat. no. 00735667, Thermo Fisher, Waltham, MA, USA) was used to perform reverse transcription.

Five hundred nanograms of the extracted total RNAs was reverse transcribed into complementary DNA (cDNA) following the manufacturer’s instructions. Random hexamers were used in a 25 µL reaction volume to carry out cDNA synthesis. SYBRGreen mix (cat.no. 4367659, Thermo Fisher Scientific, Waltham, MA, USA) was utilized for quantitative RT-PCR. A LightCycler 480 Master instrument (Roche, Basel, Switzerland) was used for the amplification (cat. no. for plates, Roche: 04729692001; cat. no. for sealing foils, Roche: 04729757001). The investigated genes are summarized in Table 2; all primers were synthesized by Invitrogen and were as follows: myosin heavy chain 1 (MHCI), myosin heavy chain 2A (MHCIIA), and myosin heavy chain 2X (MHCIIX). The 18sRNA housekeeping gene was used as the internal control (Invitrogen, Thermo Fisher Scientific, Waltham, MA, USA).

Forty-five cycles of 3 min at 95 °C were performed. The comparative Ct technique was used to determine the relative expression values for each transcript of interest. Each sample was normalized to its own internal control gene expression after being conducted in triplicate. Subsequent analysis was conducted using the mean values that were obtained.

### 4.3. In Vivo Experiments

#### 4.3.1. Body Weight Measurement

The body weight was measured at the beginning of the tamoxifen diet (at 1-month-old age) and weekly afterward until the end of the 2-month feeding period (3-month-old age) for each individual mouse in both Cre^−/−^ and Cre^+/−^ groups. In Figure 1C we present the average monthly body weight monitoring.

#### 4.3.2. Grip Strength Test

Forepaw force was measured as previously described [38]. Briefly, the animals were gently dragged away from the grip test meter by their tails after they had successfully grabbed its bar for a brief period. A computer linked to the test meter recorded the maximum force before the animal released the bar, digitizing it at a frequency of 2 kHz. Each animal underwent the test ten to fifteen times to get an averaged single data point, which was then normalized to body weight. Grip strength was assessed before, during, and after the tamoxifen diet at 1, 2, and 3 months of age, respectively.

#### 4.3.3. Rota-Rod Test

The mice were placed on a rotating cylinder (cat.no YPFB00, Dev Scientific and Engineering, India), and the duration (in seconds) spent on the rotating rod before falling was recorded. The device was set to an accelerating protocol (rpm consistently increased over time—accelerating from 4 to 300 rpm in 300 s). The mice were acclimated to the Rota-Rod before testing. The acclimatization consisted of three trials with 10 min intervals between trials at a constant speed (4 rpm) for 1 min. The performance of the mouse was tested after 30 min of rest, and the time the mouse remained on the Rota-Rod was recorded. On the day of the examination, the accelerating protocol was performed three times, and the average of the three trials was calculated for each animal. The test was carried out at various time points of the tamoxifen diet: before (1-month-old), during (2-month-old), and after (3-month-old).

### 4.4. In Vitro Experiments

Mice were anesthetized and sacrificed in compliance with the guidelines of the European Community (86/609/EEC). After CO_2_ overdose and cervical dislocation, *M. flexor digitorum* brevis (FDB), *M. extensor digitorum longus* (EDL), *M. tibialis anterior* (TA), and *M. gastrocnemius* muscles from the hind limb were dissected manually under a transmitted light microscope using thin forceps and fine precision surgical scissors.

#### 4.4.1. Isolation of Single FDB Fibers

All calcium measurements were carried out on skeletal muscle fibers from the FDB or EDL muscle of the mouse. Calcium-free Ringer’s solution (containing in mM: 136 NaCl, 5 KCl, 1 MgCl_2_, 10 HEPES; 10 glucose; pH 7.2) was used during the dissection of the muscle. Single muscle fibers from FDB were enzymatically dissociated in minimal essential media containing 0.2% type I collagenase (Sigma) at 37 °C for 45–50 min. To release single fibers, the FDB muscles were mechanically dissociated and then triturated gently in a normal Ringer solution (same as above but supplemented with 2.6 mM CaCl_2_). The isolated fibers were then placed in culture dishes and stored at 4 °C in the refrigerator until use. Only fibers with clearly visible striations and no swelling or surface membrane damage were selected for subsequent experiments.

#### 4.4.2. Mitochondrial Membrane Potential Measurement, Oxidative Stress Measurement, Confocal Microscopy, and Image Processing

Isolated FDB fibers from 3-month-old Cre^−/−^ and Cre^+/−^ mice were loaded with 20 nM tetramethylrhodamine methyl ester (TMRE) prepared in normal Ringer’s solution at room temperature for 15 min. After 20 min the excess dye was washed. To monitor TMRE fluorescence, a time series of images was acquired using a Zeiss 5Live confocal microscope (Zeiss, Oberkochen, Germany) equipped with a 20x air objective and 543 nm laser. Sequential images were taken at 10 s intervals. Twenty seconds after the start of the time series, 1 μM FCCP was perfused onto the fiber while the decay of TMRE fluorescence indicating the mitochondrial membrane potential (*ψ_m_*) dissipation was continually monitored. Using the Zeiss Zen Blue edition software (v3.12, Zeiss, Oberkochen, Germany), an arbitrarily chosen region of interest (ROI) was marked on all subsequent images, and the obtained fluorescence values were fitted with an exponential function using Equation (3) to determine the decay time (τ). The averages of the individual decay times were plotted.

In the CellRox Green experiments (cat.no. C10444, Thermo Fisher Scientific, Waltham, MA, USA), FDB fibers were loaded in normal Ringer’s solution with the dye in 1:500 dilution for 30 min at 37 °C. Excitation wavelength was at 488 nm and detection at λ > 520 nm. An arbitrary ROI (marked with a rectangle) was selected in parallel with the longitudinal axis of the fiber and the fluorescence was plotted. The ROI selection was always done in a way to avoid areas of dye accumulation (e.g., nuclei). Fluorescence was calculated similarly as described previously [66]. In short, at the peaks (I-band fluorescence, representing mitochondria (F_I-band_)) and at troughs (A-band fluorescence, representing baseline (F_A-band_)), fluorescence was measured, then the normalized mitochondrial fluorescence expressed as F_mito_ was calculated with the equation:

F_mito_ = (F_I-band_ − F_A-band_)/F_A-band_ (1)

#### 4.4.3. Assessment of Mitochondrial Oxygen Consumption Using High-Resolution Respirometry

Mitochondrial oxygen consumption (O_2_ flux) was assessed in *M. gastrocnemius* (7 mg/chamber) using High-Resolution FluoRespirometry (Oxygraph-2k, Oroboros Instruments, Innsbruck, Austria). The isolated muscles were incubated in BIOPS solution containing saponin to ensure membrane permeabilization. After stabilization of baseline respiration, we used a short and a long respirometric protocol (see below).

In the short respirometric protocol (Figure 4A), the integrity of the outer mitochondrial membrane was tested with exogenous cytochrome c (10 µM) following OXPHOS II stimulation (0.5 µM Rotenone (Rot), 10 mM succinate (S), and 2.5 mM ADP). Respiratory control ratio (RCR), an index of respiration coupled to ADP-ATP conversion, was expressed as a ratio of OXPHOS II to the ATP synthase-inhibited (oligomycin, Omy; 2.5 μM) LEAK state. The electron transport system-independent respiration (or residual oxygen consumption; ROX) was determined after complex III inhibition with antimycin A (2.5 μM).

In the long respirometric protocol (Figure 4B), complex I-linked oxidative phosphorylation (OXPHOS I) was measured in the presence of complex I-linked substrates (10 mM glutamate and 2 mM malate) and ADP (2.5 mM). Rotenone (Rot; 0.5 µM) was used to (a) inhibit complex I and (b) assess complex II-linked oxidative phosphorylation (OXPHOS II) in the presence of succinate (S; 10 mM) and adenylate. After inhibition of complex III (antimycin A; 2.5 µM), complex IV respiratory activity was measured with ascorbate (2 mM) and artificial substrate *N,N,N′,N′*-Tetramethyl-*p*-phenylenediamine dihydrochloride (TMPD; 0.5 mM). Ascorbate was added before TMPD to avoid uncontrollable autoxidation of the electron donor. Sodium azide (NaN3; 100 mM) was finally administered to block complex IV-linked mitochondrial respiration.

Both the short and long protocol measurements were performed in a Mir05 respiration medium under continuous magnetic stirring (750 rpm) at 37 °C. The DatLab 7 software (version 7.4.0.4., Oroboros Instruments, Innsbruck, Austria) was used for online display, respirometry data acquisition, and analysis.

#### 4.4.4. T-System Ca^2+^-Uptake Measurement

Mixed-gender Cre^−/−^ (n = 5) and Cre^+/−^ (n = 7) mice were investigated, and t-system uptake measurements were performed as previously described in Cully et al. [41]. Briefly, euthanasia was performed via cervical dislocation, followed by rapid dissection of the whole *M. extensor digitorum longus* (EDL) muscle, which was then placed in a glass Petri dish covered with Sylgard and submerged in paraffin oil. A bundle of fibers was exposed to a standard Ringer solution containing 2.5 mM of the low-affinity Ca^2+^-sensitive dye Rhod-5N for 10 min. Individual fibers were isolated from the Rhod-5N-loaded bundle and skinned by mechanically removing the sarcolemma. Skinned fibers were subsequently transferred to a custom-made chamber constructed on top of a 1.5 mm coverslip filled with an intracellular solution containing (in mM) 90 HEPES, 10 EGTA, 9.86 MgO_2_, 9.7 CaCO_3_, 8 Na_2_ATP, and 10 NaCP, with pH adjusted to 7.1 with KOH (yielding a free Ca^2+^ concentration of 67 nM). Adjustment of the total [Ca^2+^] and [Mg^2+^] resulted in free [Ca^2+^] concentrations of 28, 67, 200, and 1342 nM, and free [Mg^2+^] concentrations of 1 mM, respectively. The chosen calcium concentrations are a consequence of the mixing of different EGTA-buffered solutions (one low, one high in free Ca^2+^) following Cully et al. [41]. These solutions are based on earlier work by George Stephenson [67]. Depletion of the t-system was induced in a nominally Ca^2+^ and Mg^2+^-free solution with the addition of 30 mM caffeine (cat.no. C0750, Sigma). The chamber was transferred onto the stage of a confocal microscope for continuous Rhod-5N fluorescence recording. Minimal (F_min_) and maximal (F_max_) dye fluorescence were determined in the presence of maximal (5 mM) and nominally Ca^2+^-free solutions containing 25 µM of ionomycin (cat.no. I9657, Sigma) and A23187 (cat.no. C7522, Sigma). The spatially averaged real-time fluorescent signal (F) was converted to calcium concentration using the formula:

[Ca^2+^] = *β* K_D_ × (F − F_min_)/(F_max_ − F) (2)

with a K_D_ of 0.872 mM (Cully et al. [41]). Uptake fluxes were calculated as the signal’s first-time derivative.

#### 4.4.5. Resting Myoplasmic [Ca^2+^]_i_ Measurement

The resting intracellular Ca^2+^ concentration was measured using the Fura-2 AM fluorescent calcium indicator. Briefly, single FDB fibers were mounted on a glass coverslip with 5 µM Fura-2 AM dye for 1 h. Fibers were then washed with fresh normal Tyrode’s solution (137 mM NaCl, 5.4 mM KCl, 0.5 mM MgCl_2_, 1.8 mM CaCl_2_, 11.8 mM Hepes-NaOH, and 1 g/L glucose, pH 7.4) and then imaged with the CoolLED pE-340^fura^ setup (CoolLED Ltd., Hampshire, UK). The acquisition rate was set to 10 Hz. Fura-2 ratios from myoplasmic areas of interest were calculated as R = F_340_/F_380_ using Zeiss Zen Blue Edition software (Zeiss, Oberkochen, Germany) and then converted to resting free Ca^2+^ concentrations using an in situ calibration curve for Fura-2 AM according to the method of Grynkiewicz et al. [68] based on Equation (1) (see above), where *β K_d_* = 0.3.

#### 4.4.6. Whole-Cell Voltage Clamp

The experimental design was as described in our earlier report [39]. Briefly, isolated FDB fibers were voltage-clamped (Axoclamp 2B, Axon Instruments, Union City, CA, USA) and imaged using a confocal microscope (Zeiss 5 Live, Oberkochen, Germany). Fibers were dialyzed with 50 µM Rhod-2-containing internal solution (see below). The experimental temperature was 20–22 °C, and the holding potential was −80 mV. Pipette resistance varied between 1 and 2 MΩ. The experiments were performed in the presence of 10 mM EGTA so that the endogenous buffers in the removal process were almost negligible. Correction for linear capacitive currents was performed by analog compensation.

The intracellular calcium ([Ca^2+^]_i_) values were determined from the fluorescence (F) and the background fluorescence (F_0_) in line scan images analyzed by an in-house custom-made program using the following parameters taken from Royer et al. [69]: K_d Rhod-2_ = 1.58 μM and k_ON_ = 0.07 μM^−1^ ms^−1^ and k_OFF_ = 130 s^−1^. Ca^2+^-release flux was derived from cytosolic Ca^2+^ transients and subjected to a removal-model fit analysis, which calculates release flux as that necessary to account for the evolution of [Ca^2+^]_i_(t) in a single-compartment model that includes quantitatively specified processes of removal as originally described by Melzer et al. [70] and further optimized by Royer et al. [69]. In brief, the model considers that [EGTA] is sufficiently high, and thus only four forms of Ca^2+^ must be considered: free, bound to the monitoring dye, bound to EGTA, and sequestered in the SR. From the release flux (the flux exiting through the release channels), the net flux leaving the SR can be derived by subtraction of the pump-removal flux.

The evolution of SR Ca^2+^ content during repetitive stimulation in both mouse groups was calculated from the amount of calcium released minus the removal flux, which contains the uptake of Ca^2+^ into the SR by the SERCA pump.

To estimate the average amount of calcium in the SR from the depolarizing train, single-exponential functions were fitted to the eight points of the series. The exponential decay was assessed by the equation below:

y = y_0_ + ae^−bx^ (3)

where x is the number of tetanic pulses applied, b is the time constant of SR depletion, and a is the remaining SR calcium content.

External bath solution (in mM): 140 TEA-CH_3_SO_3_, 1 CaCl_2_, 3.5 MgCl_2_, 10 Hepes, 1 4-AP, 0.5 CdCl_2_, 0.3 LaCl_3_, 0.001 TTX (citrate), and 0.05 BTS (N-benzyl-p-toluene sulphonamide; Sigma-Aldrich). pH was adjusted to 7.2 with TEA-OH, and osmolality was adjusted to 320 mOsm with TEA methanesulfonate.

Internal (pipette) solution (in mM): 110 N-methylglucamine, 110 L-glutamic acid, 10 EGTA, 10 Tris, 10 glucose, 5 Na ATP, 5 phosphocreatine Tris, 0.1 Rhod-2, 3.56 CaCl_2_, and 7.4 mM MgCl_2_ were added for a nominal 1 mM [Mg^2+^] and 100 nM [Ca^2+^]. pH was set to 7.2 with NaOH, and osmolality to 320 mOsm with N-methylglucamine.

### 4.5. Quantification and Statistical Analysis

Pooled data were expressed as the mean ± standard error of the mean (SEM). The differences between Cre^−/−^ and Cre^+/−^ mice were assessed using one-way analysis of variance (ANOVA) and all pairwise Bonferroni’s multiple comparison methods using the statistical program Prism 9 (GraphPad Software, San Diego, CA, USA). Student’s *t*-test was used to test the significance, and a *p*-value of less than 0.05 was considered statistically significant. G-Power (version 3.1.9.7) software was used to estimate the number of specimens included in the in vivo experiments (α = 0.05; assumed effect size: 1; calculated power: 0.9548089).

## 5. Conclusions

To develop effective therapies for conditions with defective endocannabinoid system (ECS) activity, it is essential to understand the skeletal ECS’s function in health and disease. Here, we present a detailed functional and molecular characterization of a transgenic mouse model with skeletal muscle-specific *Cnr1* genetic ablation. Our in vivo results demonstrate that CB_1_R knockdown impairs motor coordination and grip strength in mice. Furthermore, at the cellular level, we observed modestly altered calcium homeostasis and significantly decreased mitochondrial function (decreased complex IV activity). Therefore, we propose that the ECS (particularly CB_1_R) plays a key role in physiological muscle force generation and in maintaining cellular and mitochondrial homeostasis and function.

## Figures and Tables

**Table 1:** List if antibodies used in Western blot analysis.

Name	Cat. no.	Company	Dilution
anti-Orai1	MA5-15777	Invitrogen	1:500
anti-STIM1	AB-9870	Sigma	1:1000
anti-PMCA	5F10	Abcam *	1:1000
anti-VDAC	MA5-35349	Invitrogen	1:1000
anti-MICU1	PA5-77364	Invitrogen	1:200
anti-Drp1	C6C7	Cell Signaling Technology **	1:1000
anti-Mfn2	D2D10	Cell Signaling Technology	1:1000
anti-Opa1	MA-16149	Invitrogen	1:1000
anti-α-actinin	sc-166524	Santa Cruz Biotechnology	1:1000
anti-tubulin	T5168	Sigma	1:4000

* Abcam, Cambridge, UK; ** Cell Signalling Technology, Danvers, MA, USA.

**Table 2:** Primer sequences used for identifying myosin heavy chain isoforms in muscle samples.

Name	Forward Primer	Reverse Primer
MHCI	5′-GAGTAGCTCTTGTGCTACCCAGC-3′	5′-AATTGCTTTATTCTGCTTCCACC-3′
MHCIIA	5′-GCAAGAAGCAGATCCAGAAAC-3′	5′-GGTCTTCTTCTGTCTGGTAAGTAAGC-3′
MHCIIX	5′-GCAACAGGAGATTTCTGACCTCAC-3′	5′-CCAGAGATGCCTCTGCTTC-3′
18s RNA	5′-GGGAGCCTGAGAAACGGC-3′	5′-GGGTCGGGAGTGGGTAATTTT-3′

## Data Availability

Data will be made available on reasonable request.

## Supplementary Materials

The following supporting information can be downloaded at: https://www.mdpi.com/article/10.3390/ijms26115291/s1.
