# Supplementary material for: Physiological Muscle Function Is Controlled by the Skeletal Endocannabinoid System in Murine Skeletal Muscles
Source: Int J Mol Sci. 2025 May 30;26(11):5291. doi: 10.3390/ijms26115291 (PMC12155153; doi:10.3390/ijms26115291)
Supplement: Supplementary file 1 [file ijms-26-05291-s001.zip › ijms-3596257-supplementary.pdf]

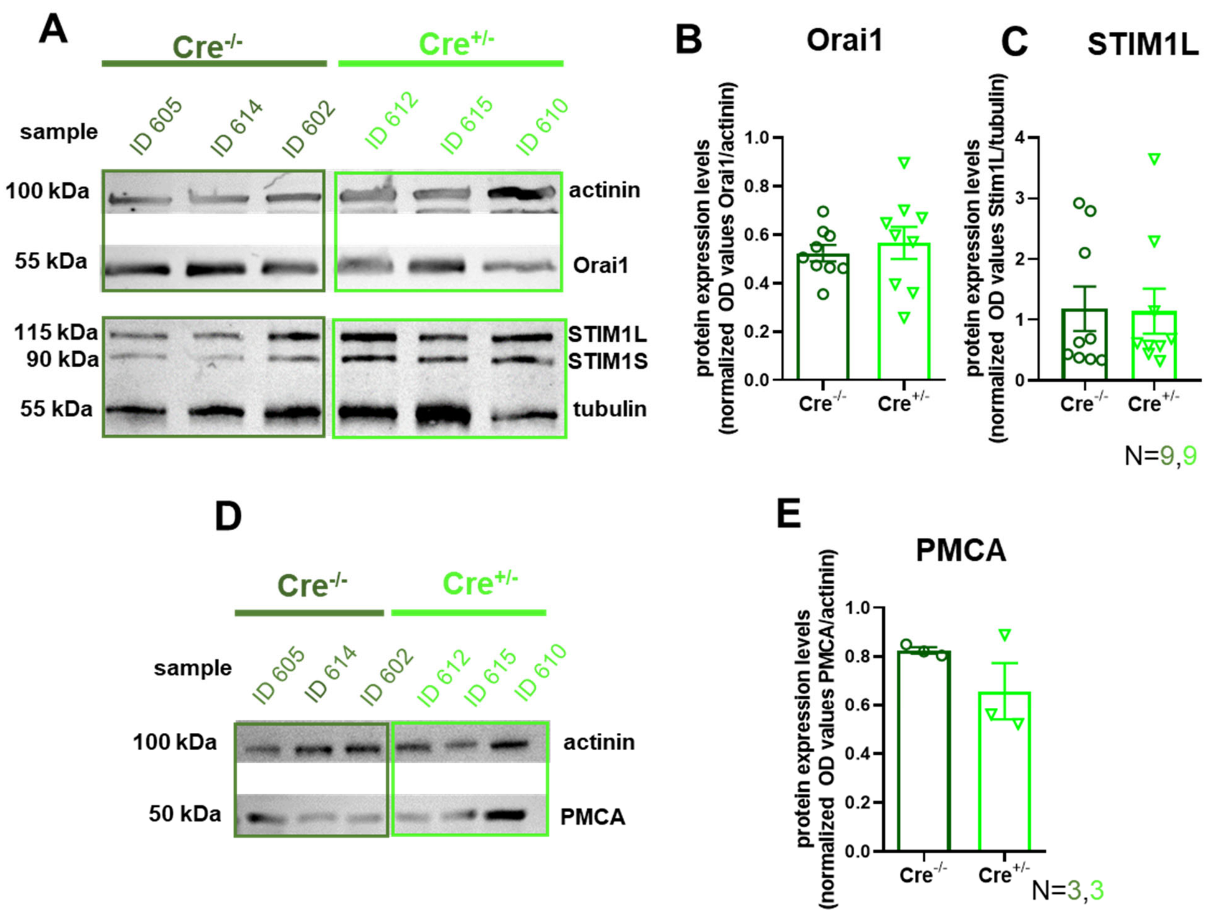

Supplementary Figure S1. Western Blot analysis of SOCE partner proteins and PMCA. (**A**) Representative Western Blot gels probed for the SOCE partners: STIM1 and Orai1. Actinin or tubulin were used as internal controls. 3 animals were examined for each group. (**B**,**C**) Bar graphs plotted from 9 individual experiments show the expression of STIM1 and Orai1 in TA muscles after 2 months of Tamoxifen feeding. Each bar is the mean ± S.E.M. Both STIM1 short (S) and long (L) isoforms were detected but in panel B we show only STIM1L normalized to tubulin. Orai1 was normalized to actinin. (**D**,**E**) Representative Western Blot gel and bar graph representation following densitometry obtained from 3 independent experiments show unchanged PMCA expression in TA muscles in 3 $Cre^{-/-}$ and 3 $Cre^{+/-}$ mice. Abbreviations: SOCE, store-operated calcium entry; TA, *m. tibialis anterior*; PMCA, plasma membrane calcium ATPase.

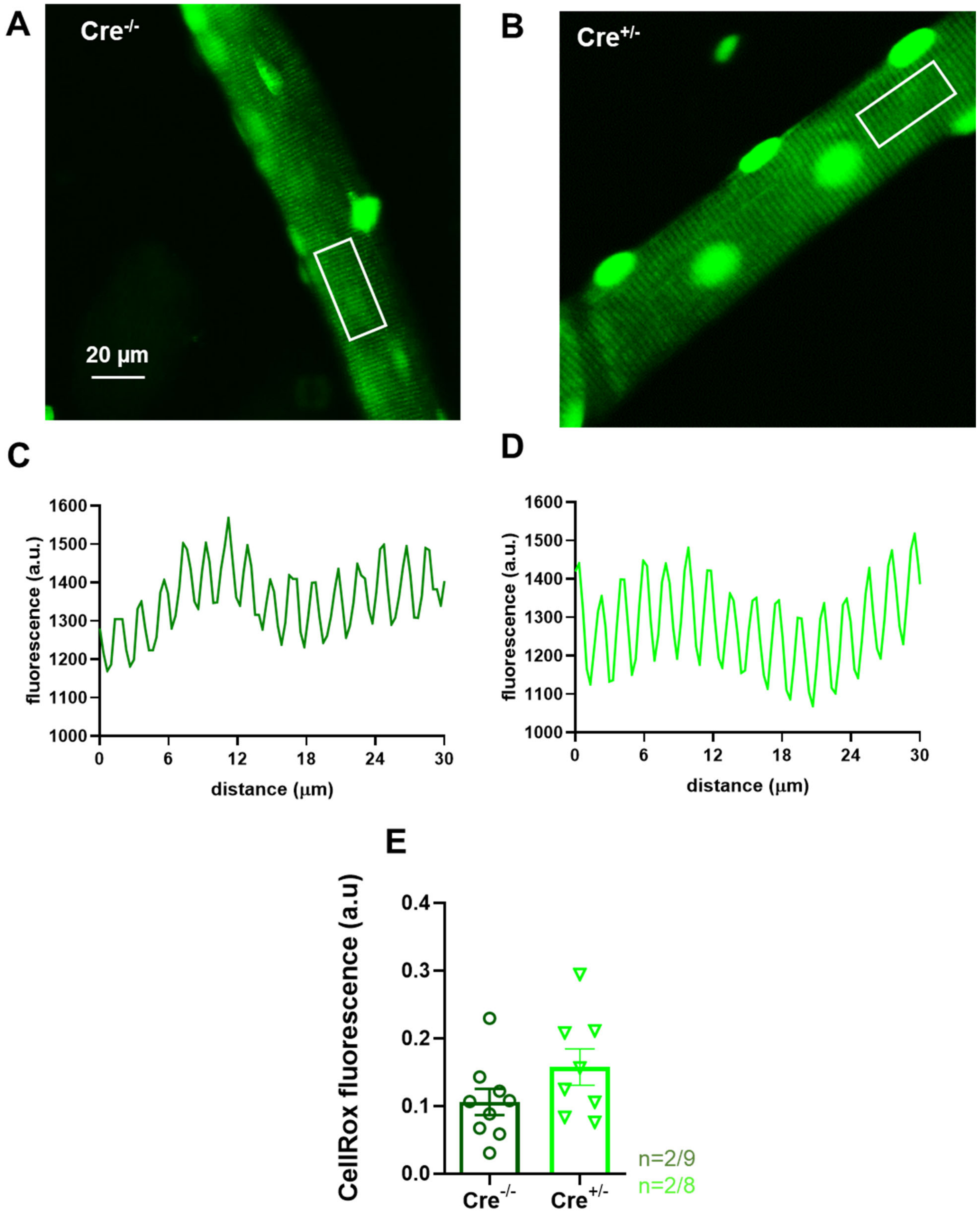

Supplementary Figure S2. Oxidative stress measurement with CellRox Green in FDB fibers. (A,B) Fluorescence intensity of a Cre$^{-/-}$ and a Cre$^{+/-}$ FDB fiber at rest, loaded for 30 min at 37°C with CellRox

Green (1:500 dilution), then excited with a confocal laser microscope at 488 nm and fluorescence detected at λ>520 nm. (**C**,**D**) The background corrected fluorescence values were averaged over the area marked with a rectangle selected in parallel with the longitudinal axis of the fiber. Note the periodic increase and decrease in fluorescence representing the sarcomeric pattern of dye distribution. (**E**) Average CellRox Green fluorescence reveals no change upon CB_1R downregulation. Results presented are from 9 and 8 fibres, from 2 $Cre^{-/-}$ and 2 $Cre^{+/-}$ animals, respectively.

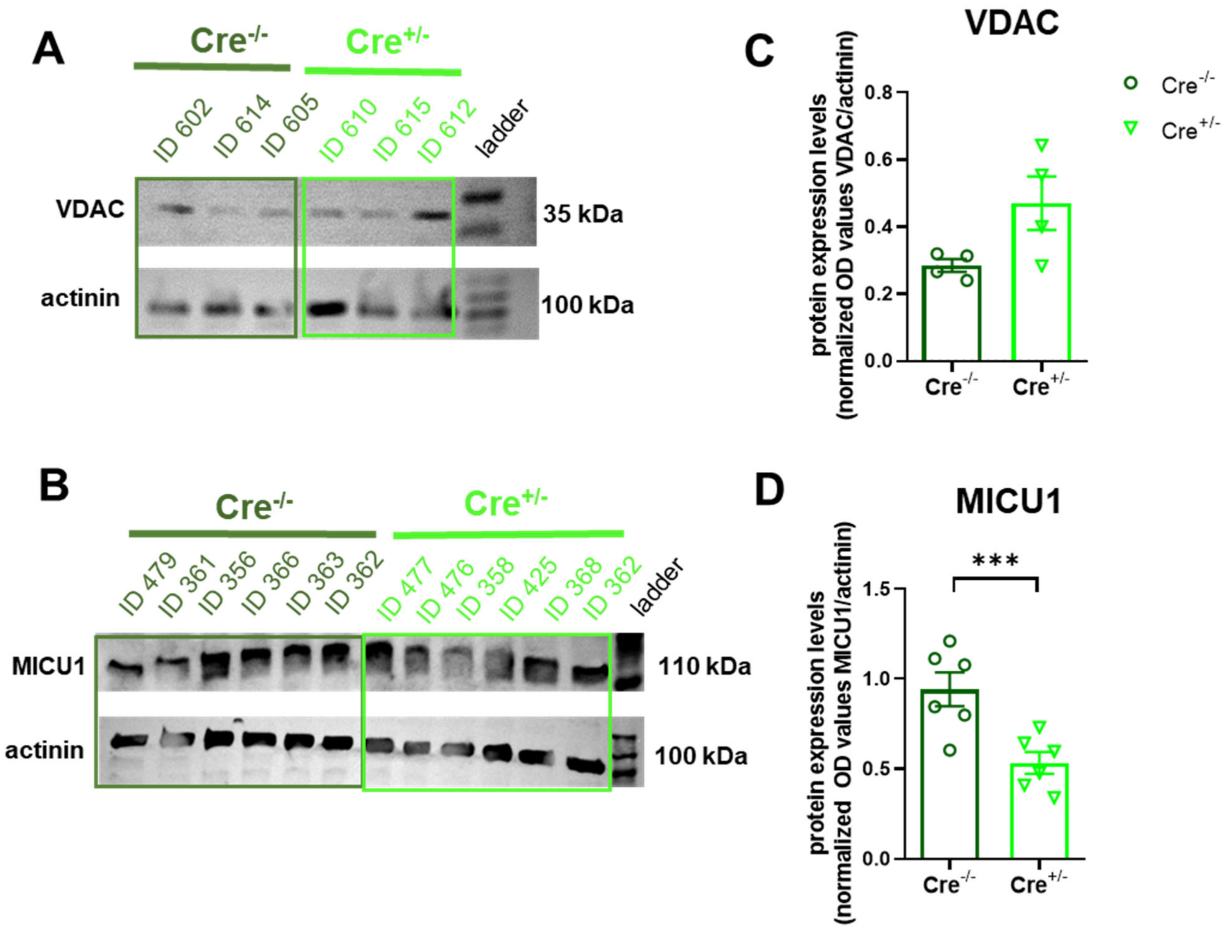

Supplementary Figure S3. Examination of mitochondrial proteins VDAC and MICU1 in TA muscles. Representative Western Blot images were probed for VDAC (**A**) and MICU1 (**B**). (**C**) The bar diagrams compiled from 4 independent experiments performed on 3 $Cre^{-/-}$ and 3 $Cre^{+/-}$ specimens show a slight but non-significant increase in relative protein expression for VDAC as normalized to actinin (**D**) MICU1 was significantly downregulated in $Cre^{+/-}$ muscles. Data is from 6 independent experiments performed on 6 $Cre^{-/-}$ and 6 $Cre^{+/-}$ specimens. *** indicates statistical significance at $p<0.0001$. Abbreviations: VDAC, voltage-dependent anion channel; MICU1, mitochondrial calcium protein 1.

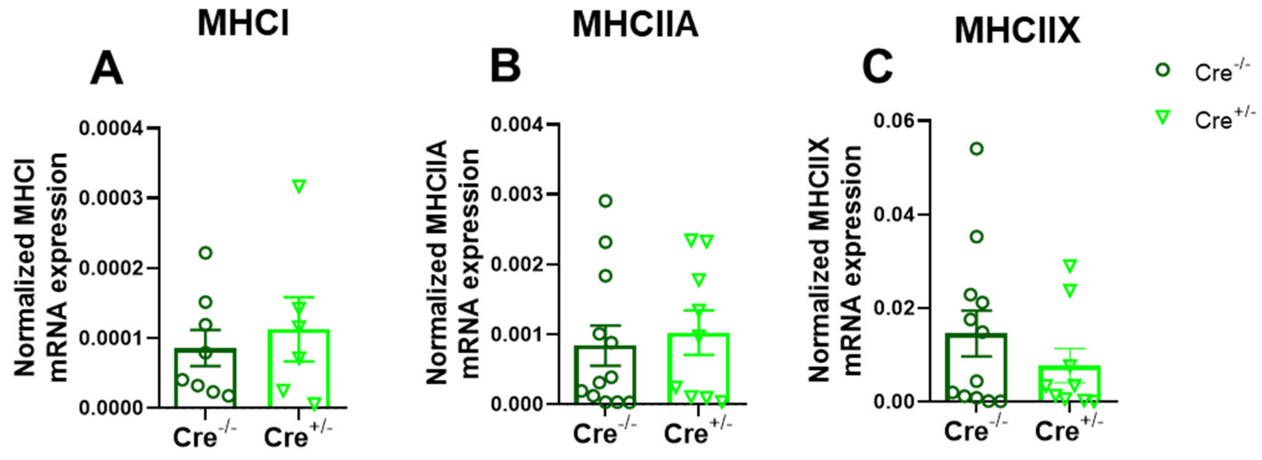

Supplementary Figure S4. No fiber type change was detected on mRNA level upon CB_1R downregulation. We have analyzed both the slow (MHCI) (**A**) and fast myosin heavy chains (MHCIIA) (**B**) and (MHCIIX) (**C**) in samples prepared from TA muscles. Although we detected by trend decreased MHCIIX mRNA levels in $Cre^{+/-}$ specimens compared to the control $Cre^{-/-}$, the difference was not significant. Data was obtained from n = 3 technical triplicates from 4 $Cre^{-/-}$ and 3 $Cre^{+/-}$ biological samples, respectively. 18sRNA was used as housekeeping gene to obtain normalized data.

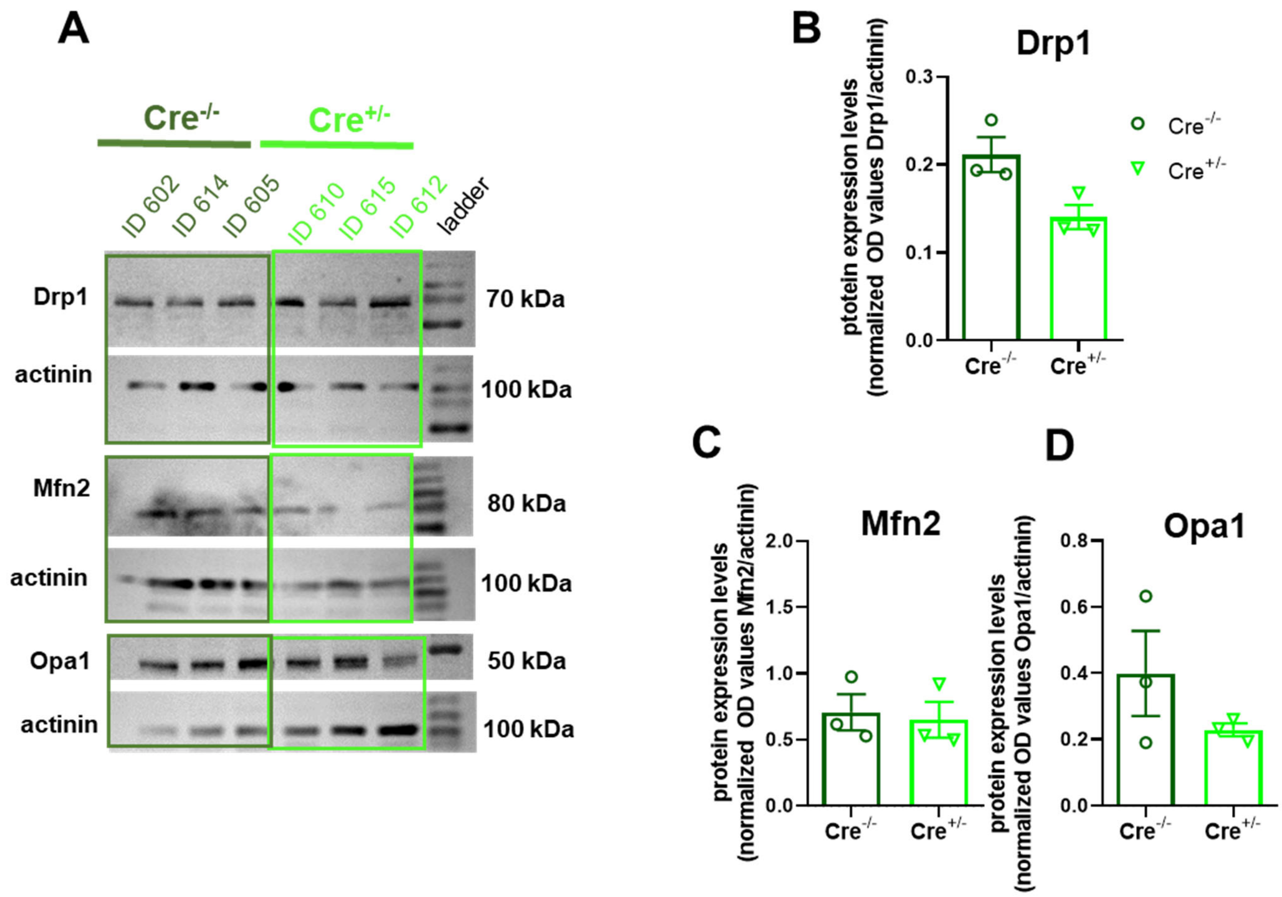

Supplementary Figure S5. Mitochondrial dynamics-related proteins show no change in Cre$^{+/-}$ mice. (**A**) Representative Western Blot images probed for Drp1, Mfn2, and Opa1. The bar diagrams show averaged relative protein expression for Drp1 (**B**)**,** Mfn2 (**C**), and Opa1 (**D**) as normalized to actinin. 3-5 independent experiments were performed investigating 3 Cre$^{-/-}$ and 3 Cre$^{+/-}$ animals. Abbreviations: Drp1, dynamin related protein 1; Mfn2, mitofusin 2; Opa1, optic atrophy 1.
